# Preparation, Optimization, and In-Vitro Evaluation of Brusatol- and Docetaxel-Loaded Nanoparticles for the Treatment of Prostate Cancer

**DOI:** 10.3390/pharmaceutics16010114

**Published:** 2024-01-16

**Authors:** Tayo Alex Adekiya, Madison Moore, Michael Thomas, Gabriel Lake, Tamaro Hudson, Simeon K. Adesina

**Affiliations:** 1 Department of Pharmaceutical Sciences, College of Pharmacy, Howard University, Washington, DC 20059, USA; 2 Department of Biology, Howard University, Washington, DC 20059, USA; 3 Cancer Center, Howard University, Washington, DC 20059, USA

**Keywords:** prostate cancer, docetaxel, brusatol, nanoparticles, cell cycle, caspase activity, survivin

## Abstract

Challenges to docetaxel use in prostate cancer treatment include several resistance mechanisms as well as toxicity. To overcome these challenges and to improve the therapeutic efficacy in heterogeneous prostate cancer, the use of multiple agents that can destroy different subpopulations of the tumor is required. Brusatol, a multitarget inhibitor, has been shown to exhibit potent anticancer activity and play an important role in drug response and chemoresistance. Thus, the combination of brusatol and docetaxel in a nanoparticle platform for the treatment of prostate cancer is expected to produce synergistic effects. In this study, we reported the development of polymeric nanoparticles for the delivery of brusatol and docetaxel in the treatment of prostate cancer. The one-factor-at-a-time method was used to screen for formulation and process variables that impacted particle size. Subsequently, factors that had modifiable effects on particle size were evaluated using a 2^4^ full factorial statistical experimental design followed by the optimization of drug loading. The optimization of blank nanoparticles gave a formulation with a mean size of 169.1 nm ± 4.8 nm, in agreement with the predicted size of 168.333 nm. Transmission electron microscopy showed smooth spherical nanoparticles. The drug release profile showed that the encapsulated drugs were released over 24 h. Combination index data showed a synergistic interaction between the drugs. Cell cycle analysis and the evaluation of caspase activity showed differences in PC-3 and LNCaP prostate cancer cell responses to the agents. Additionally, immunoblots showed differences in survivin expression in LNCaP cells after treatment with the different agents and formulations for 24 h and 72 h. Therefore, the nanoparticles are potentially suitable for the treatment of advanced prostate cancer.

## 1. Introduction

Despite advances in treatment and improved diagnostic tools, the incidence of prostate cancer has increased by 3% per year overall and by 5% per year for advanced prostate cancer since 2014 in the United States. Additionally, prostate cancer is the second leading cause of cancer death in American men [1]. Currently, it is estimated that about 12.9% of men will be diagnosed with the disease during their lifetime [2]. Treatment is largely dependent on the stage and grade of cancer, and some men eventually develop metastatic prostate cancer with androgen deprivation therapy (ADT) as the standard of care [3]. Disease progression and resistance to ADT ultimately leads to the development of metastatic castration-resistant prostate cancer (mCRPC). mCRPC is defined as prostate cancer characterized by clinical, radiographic, or biochemical progression despite castration levels of serum testosterone [4]. Metastatic castration-resistant prostate cancer is often treated using docetaxel and prednisone as first-line chemotherapy [5]. Docetaxel (Taxotere) is a potent, first-line chemotherapeutic drug that was approved by the US Food and Drug Administration (FDA) in 2004 for patients with castration-resistant prostate cancer (CRPC) that has progressed despite hormone therapy. The main mechanism of action of docetaxel is binding to the tubular protein, specifically to the β-tubulin subunit, preventing microtubule depolymerization leading to G2/M phase (mitotic) arrest and apoptosis. However, a vast majority of patients treated with docetaxel develop resistance to the drug in addition to other reported toxicity [6,7,8]. Thus, to overcome the challenges associated with docetaxel use and to improve therapeutic efficacy in heterogenous prostate cancer, the use of agents that inhibit multiple targets or the use of multiple agents that can destroy different subpopulations of the tumor leading to greater antitumor efficacy have been exploited.

The development of multitarget inhibitors such as dual-target inhibitors or dual-target drugs with synergistic effects is gaining ground in tumor treatment [8]. These novel agents are designed to simultaneously target and inhibit multiple key tumor survival pathways, hold the potential to overcome chemoresistance, and inhibit the capacity of the tumor cell to upregulate survival pathways thereby improving therapeutic efficacy compared to single-target agents. Closely related to dual-target drugs, the combination of two or more anticancer drugs has been reported to be superior to monotherapy for many cancers by their capacity to improve the rates and durability of response to therapy [9]. This superior response has been ascribed to the ability of combination therapy to facilitate synergism and address the issues of tumor homogeneity. Like dual-target drugs, combination therapies can overcome resistance, enhance response compared to monotherapy, and, in addition, reduce dose-limiting single agent toxicity [10,11,12].

Brusatol, a quassinoid isolated from *Brucea javanica* (L.) is a multitarget inhibitor which has been shown to exhibit potent anti-cancer activity as a protein synthesis inhibitor and, specifically, as an inhibitor of the nuclear factor erythroid 2-related factor 2 (Nrf2) pathway [13,14]. Activation of the Nrf2 pathway has been reported to be involved in the induction of drug efflux pumps and metabolizing enzymes and thus plays an important role in drug response and chemoresistance [15]. The Nrf2 pathway also plays a crucial role in the regulation of cellular defense against xenobiotic and oxidative stress and inflammation, which are implicated in the development and progression of prostate cancer [13,16,17]. Several reports have shown that brusatol elicits potent anticancer activity in preclinical studies using several cancer models such as breast cancer, hepatocellular carcinoma, lung cancer, pancreatic cancer, and prostate cancer [16,17,18,19,20,21,22]. Thus, the combination of brusatol with docetaxel in the treatment of prostate cancer is expected to produce synergistic effects as a result of its potent multitarget anticancer effects and its potential to inhibit the mechanisms that facilitate resistance to docetaxel.

The use of nanotechnology in the design and development of drug delivery systems has revolutionized the clinical treatment of cancers. Nanoparticle-based drug delivery systems have emerged as a promising approach for enhancing the efficacy and safety of anticancer drugs [17,23]. Poly (lactic-co-glycolic acid) (PLGA) nanoparticles have gained significant attention due to their biocompatibility, biodegradability, and ability to encapsulate drugs with widely ranging physicochemical properties [24,25]. In addition, PLGA nanoparticle-based drug delivery systems offer the advantages of controlled drug release, improved drug stability, and prolonged circulation time [24,25]. A major advantage of nanoparticle drug delivery is the preferential accumulation of drug-loaded nanoparticles in the tumor via the enhanced permeability and retention effect leading to improved therapeutic efficacy and reduced side effects [10]. Thus, combination nanotherapeutics is expected to combine the advantages of nanotechnology with those of combination therapy.

The efficiency of tumor accumulation of polymeric nanoparticles in vivo is largely impacted by particle size and surface characteristics of the formulations [26]. Thus, the design of nanoparticle platforms with critical quality attributes to avoid clearance by the cells of the reticuloendothelial system (RES) and preferentially accumulate in the tumor is essential. To avoid RES uptake, the fabrication of nanoparticles with particle sizes of less than 200 nm in diameter with stealth property is preferred [26]. In this work, a full factorial statistical experimental design was used to develop a model for the optimization of nanoparticle size with the goal of particle size minimization after screening for formulation and process variables that impact particle size using the one-factor-at-a-time method. Optimization of the size of nanoparticle formulations has traditionally been carried out by altering one factor or variable at a time. This laborious and time-consuming approach also limits the understanding of how interactions among the different formulation and process variables impact particle size [27]. Design of experiments (DOE) is preferred for model generation and optimization because it simultaneously examines all the variables and the effect of the interaction of factors. Hence, the use of the full factorial design helps to overcome the challenges of the one-factor-at-a-time approach by collectively optimizing all the selected or independent variables at the same time and minimizes the number of experiments required for optimization [28]. In addition, the model obtained from these studies can further be used for the optimization and prediction of the nanoparticle size [29].

This article provides an overview of the fabrication, optimization, characterization, and in vitro biological evaluations of docetaxel- and brusatol-loaded nanoparticles. Herein, we evaluate the potential of combination anticancer agents, docetaxel and brusatol, loaded in a polymeric nanoparticle platform for the treatment of prostate cancer.

## 2. Materials and Methods

### 2.1. Materials

Methoxy-terminated polyethylene glycol-poly (lactide-co-glycolide) (mPEG-PLGA) (lactic acid:glycolic acid 1:1) was purchased from Polyscitech^®^ (Akina Inc., West Lafayette, IN, USA). Polyvinyl alcohol (PVA 99^+^% hydrolyzed, MW 89,000–98,000) was purchased from Millipore Sigma (St. Louis, MO, USA). Brusatol was purchased from Carbosynth (San Diego, CA, USA). Docetaxel was purchased from Millipore Sigma (St. Louis, MO, USA). Solvents were purchased from Sigma-Aldrich and used as received. CyQUANT™ XTT Cell Viability Assay, Cell Event™ Caspase-3/7 Green Flow Cytometry Assay Kit, FxCycle™ PI/RNase Staining Solution, penicillin-streptomycin (Gibco) were purchased from Thermo Fisher Scientific (Waltham, MA, USA). RPMI 1640 and Fetal bovine serum (FBS) were obtained from ATCC (Manassas, VA, USA).

### 2.2. Methods

#### 2.2.1. Preparation and Optimization of Stealth Blank Nanoparticles

For one-factor-at-a-time experiments and full factorial statistical experimental design experiments, blank nanoparticles were prepared by the oil-in-water (o/w) emulsification solvent evaporation method using a modified published method [30]. The amounts and composition of the different formulations used for one-factor-at-a-time experiments are shown below (Table 1). For the full factorial design, the formulations generated using Minitab^®^ (Minitab LLC, State College, PA, USA) were prepared. Briefly, the desired amount of mPEG-PLGA was dissolved in the selected organic phase and emulsified in an aqueous solution of polyvinyl alcohol (PVA) in an ice bath using a probe sonicator. The emulsion obtained was poured into a 250 mL beaker containing 30 mL of deionized water under a fume hood with continuous magnetic stirring to evaporate the organic phase. Nanoparticles were obtained by centrifugation and washed thrice with deionized water during centrifugation/redispersion cycles. Nanoparticles were recovered by centrifugation at 20,000 rpm for 20 min at 4 °C. The recovered nanoparticles were freeze dried to obtain white powder.

#### 2.2.2. Initial Screening Using the One-Factor-at-a-Time Method

Using the one-factor-at-a-time approach, nanoparticle formulations were prepared using the emulsification-solvent evaporation method by varying each of the identified factors while keeping the others constant. Particle sizes were obtained and the factor that gave the smallest particle size was selected and used in other formulations to evaluate the effect of a single factor on particle size as shown in Table 1:

**Table 1:** Initial factors screened using the one-factor-at-a-time approach.

Factors	Variables
Solvent composition (Organic phase)	Ethyl acetate (EA):Dimethylformamide (DMF)
	Ethyl acetate (EA):Dimethyl sulfoxide (DMSO)
	Ethyl acetate (EA):Acetonitrile
	Ethyl acetate (EA):Acetone
	Ethyl acetate (EA):DMSO:Acetone
Solvent ratio (EA: other solvent) (Total volume = 2 mL)	1.2:0.8
	1.4:0.6
	1.6:0.4
	1.8:0.2
mPEG-PLGA concentration (mg/2 mL)	20, 50, 75, 100, 150
Organic: Aqueous ratio	2:8
	2:10
	2:12
	2:14
	2:16
Polyvinyl alcohol concentration (%)	0.25, 0.5, 1.0, 2.0, 3.0
Sonication pulse rate (on:off)	7:3, 8:2; 9:1
Amplitude (%)	25, 30, 35, 40
Sonication time (minutes)	2.5, 5, 7.5, 10

#### 2.2.3. Full Factorial Statistical Experimental Design

For this stage, factors observed to influence the reduction of nanoparticle size in the preliminary screening stage were selected. These factors or independent variables (polymer concentration, organic to aqueous ratio, PVA concentration, and amplitude) were varied on two levels (determined from earlier studies) in a 2^4^ full factorial experimental design. Two replicates were conducted for each formulation with 5 center points using Minitab^®^ software version 21.1.0.0 (Minitab LLC, State College, PA, USA) to generate 37 randomized nanoparticle formulations. The objective of this stage was to develop a model for particle size minimization (dependent or outcome variable). To check the validity of the model, the response optimizer feature of the Minitab^®^ software was used for the prediction of particle size and nanoparticle size optimization. Confirmatory experiments were carried out in triplicate.

### 2.3. Preparation of Stealth Brusatol- and Docetaxel-Loaded Nanoparticles

The encapsulation of varying amounts of drugs (brusatol: docetaxel), using different ratios of the total amount of drugs to polymer was carried out using the oil-in-water emulsification solvent evaporation method as described for blank nanoparticles using the optimized formulation with slight modifications (Table 2).

To prepare the nanoparticles, brusatol was dissolved in DMSO (0.2 mL) with vigorous vortexing. After a clear solution was obtained, acetone (0.2 mL) was added, and the resulting clear solution was added to docetaxel and vortexed. Following the formation of a clear drug solution, the polymer was added followed by vortexing until complete dissolution. Ethyl acetate (1.6 mL) was then added to obtain a clear drug and polymer solution (2 mL) as the organic phase. The organic phase was emulsified in the aqueous phase (12 mL of 0.5% PVA solution in water) using a probe sonicator (Vibra-Cell; Model VC 750, Sonics and Materials, Newton, CT, USA) for 10 min at 26% amplitude using a pulse cycle of 8 s on and 2 s off. The drug-loaded nanoparticles were recovered and lyophilized as described for blank nanoparticles above. Experiments were conducted in triplicate.

#### 2.3.1. Evaluation of Particle and Zeta Potential

The particle size and size distribution of the different batches of blank and drug-loaded nanoparticles were determined by dynamic light scattering (DLS) using a 90 Plus particle size analyzer (Brookhaven Instruments Corp., Holtsville, NY, USA). Each measurement was carried out by dispersing 200 μL of the prepared nanoparticle suspension in 4 mL of DI water and the resulting suspension was filtered through a 5 µm Millex^®^ SV syringe filter (Merck Millipore Ltd., Tulla green, Carrigtwohill, Cork, Ireland).

The zeta potential was determined using a 90 Plus particle size analyzer (Brookhaven Instruments Corp., Holtsville, NY, USA). Briefly, 5 mg of freeze-dried nanoparticles were suspended in 5 mL of deionized water. It was briefly sonicated for 30 s and filtered through a 5 µm Millex^®^ SV syringe filter.

Measurements for particle size and zeta potential analyses were performed in triplicate at 25 °C. A measure of the particle size distribution is given by the polydispersity index (PDI).

#### 2.3.2. Morphological Studies

The structural morphology of the nanoparticles was analyzed using transmission electron microscopy (TEM) (FEI Talos^TM^ F200X Microscope) (Thermo Fisher Scientific Inc., Waltham, MA, USA). Negative staining of the nanoparticle suspension followed by TEM imaging of the dry grids afforded the images.

#### 2.3.3. Infrared Spectroscopy Analysis

Fourier transform infrared spectroscopy (FT-IR) analysis was carried out to evaluate potential interactions between each drug and polymer and to qualitatively evaluate the efficiency of encapsulation of both drugs using a Spectrum 100 Fourier Transform Infrared (FT-IR) spectrophotometer (Perkin Elmer, Shelton, CT, USA). FT-IR spectra were acquired for blank nanoparticles, brusatol- and docetaxel-loaded nanoparticles, pure brusatol, pure docetaxel, PLGA-PEG polymer, physical admixture of polymer and drugs, and overlaid.

#### 2.3.4. Drug Content Determination

The weight percent of brusatol and docetaxel in the optimized nanoparticle formulation was quantified by High-Performance Liquid Chromatography (HPLC) from standard calibration curves of pure drugs. For these analyses, a known amount of the freeze-dried brusatol- and docetaxel-loaded nanoparticles was dissolved in 3 mL of acetonitrile and filtered through a 0.2 μm syringe filter. The amount of drug in the solution was quantified using a validated HPLC method on an Agilent series 1100 HPLC equipped with a Zorbax Eclipse plus C 18 column kept at 37 °C. A simple gradient method with mobile phase consisting of deionized water (A) and acetonitrile (B) was run from 10–99% (B) over a period of 12 min at a flow rate of 1 mL/min. The quantitation of brusatol and docetaxel were carried out using a diode array detector at 235 nm and 230 nm, respectively. The percent drug loading was calculated using the equation below:

$$Drug\;loading\;(\%)=\frac{Weight\;of\;each\;drug\;incorporated\;in\;nanoparticle\times 100}{Total\;weight\;of\;nanoparticle}$$

#### 2.3.5. Release Profile of Docetaxel- and Brusatol-Loaded Nanoparticles

The release profile of brusatol and docetaxel from the nanoparticles was evaluated using a modification of the dialysis bag method as previously described [31]. Briefly, 10 mg of the freeze-dried nanoparticles was dispersed in 3 mL phosphate buffered saline (PBS) in a dialysis bag (molecular weight cut off of 12,000–14,000). The dialysis bag was immersed in a 15 mL Eppendorf tube containing PBS to completely cover the dialysis bag (total volume of PBS = 13 mL) and the tube was clamped to a Fisherbrand rotator shaker with 360° rotation operated at 10 rpm in an oven maintained at 37 °C. Sampling of the release medium was carried out at different time intervals (0, 0.5, 1, 2, 4, 8, 24, 48 h) and replaced with an equal volume of PBS to maintain sink conditions. The sample was diluted with acetonitrile, filtered through a 0.2 µm syringe filter, and analyzed using the HPLC method earlier described. The experiments were conducted in triplicate.

### 2.4. Cell Culture Experiments

All studies involving cell cultures were carried out using LNCaP (PSA-expressing prostate cancer cell line) and PC-3 (non-PSA-expressing prostate cancer cell line) as model prostate cancer cell lines.

LNCaP and PC-3 were obtained from the College of Medicine, Howard University. The prostate cancer cell lines were cultured in RPMI 1640 (ATCC, Manassas, VA, USA) supplemented with 10% FBS (ATCC, Manassas, VA, USA) and 1% penicillin-streptomycin (Gibco, Thermo Fisher Scientific, Waltham, MA, USA). The cells were maintained at 37 °C in a humid atmosphere containing 5% CO_2_.

#### 2.4.1. Combination Index Determination

The in vitro cytotoxicity of pure docetaxel and brusatol on LNCaP and PC-3 cells was investigated using the CyQUANT XTT cell viability assay (Invitrogen, Waltham, MA, USA). LNCaP cells (6000 cells/well) and PC-3 cells (4000 cells/well) were seeded in 96-well plates and allowed to adhere overnight. The culture media was then replaced with 100 µL of culture media containing different concentrations (5 nM, 10 nM, 20 nM, 40 nM, 80 nM, 160 nM) of either single drugs or a combination of the drugs (brusatol and docetaxel). At 72 h post treatment, the XTT cell viability assay was carried out per manufacturer protocol. Absorbance was read at a wavelength of 450 nm on a Biotek ELx808 absorbance microplate reader (Lonza, Walkersville, MD). Results are presented as percent viability normalized to controls. Data represents the mean ± SD of four replicates per concentration tested.

#### 2.4.2. Viable Cell Count and In Vitro Cytotoxicity

The viable cell count was performed by seeding LNCaP and PC-3 cells in 12-well plates at 1.5 × 10^5^ cells/mL. After 24-h incubation, the cells were treated with different concentrations of pure docetaxel (30 nM, 60 nM), pure brusatol (20 nM, 40 nM), combination drug solution (20 nM brusatol + 30 nM docetaxel; 40 nM brusatol + 60 nM docetaxel), and nanoparticle formulations containing same drug concentrations as combination drug solutions and incubated for 24 h and 72 h. The controls used include blank nanoparticles at concentrations equivalent to the dual-drug loaded nanoparticles, DMSO at the same concentration used for dissolving the pure drugs and cell culture media. Cell counts at 24 h and 74 h for the different treatments was achieved by mixing 10 µL of cell suspension with 10 µL of 0.4% trypan blue staining solution followed by the counting of live cells using an automated cell counter (Countess II^TM^–Thermo Fisher Scientific, Waltham, MA, USA). The experiments were conducted in triplicate. The percentage of viable cells was determined by comparing the mean cell count of live cells in the treated samples with that of the cell culture media controls using the equation below:

$$\%\;Viable\;cell=\frac{Count\;of\;live\;cells\;in\;treated\;well\times 100}{Count\;of\;live\;cells\;in\;media\;treated\;control\;wells}$$

For in vitro cytotoxicity evaluations, the approach used in combination index determination was employed. Cells were treated with cell culture media containing different concentrations of drugs (docetaxel/brusatol—5/3.41 nM; 10/6.82 nM; 20/13.65 nM; 40/27.35 nM; 80/54.6 nM 160/109.2 nm) based on the nanoparticle drug loading determined by HPLC. Viability was determined using the MTS assay per manufacturer protocol.

#### 2.4.3. Flow Cytometry Studies

##### Cell Cycle Arrest Analysis

This was carried out using a modified published method [32]. LNCaP cells were seeded in 12-well plates at 2 × 10^5^, 1.5 × 10^5^, and 1 × 10^5^ cells/mL for the evaluation of cell cycle arrest at 24 h, 72 h, and 120 h, respectively. Similarly, PC-3 cells were seeded in 12-well plates at 1 × 10^5^, 7.5 × 10^4^, and 5 × 10^4^ cells/mL for the evaluation of cell cycle arrest at 24 h, 72 h, and 120 h, respectively. After 24-h incubation, the cells were treated with different concentrations of docetaxel solution (60 nM), brusatol solution (40 nM), combination drug solution (40 nM brusatol + 60 nM docetaxel), and nanoparticle suspension containing same drug concentrations as the combination drug solution and incubated for 24 h, 72 h, and 120 h. The controls used included blank nanoparticles at concentrations equivalent to the dual-drug loaded nanoparticles, DMSO at the concentration used for dissolving the pure drugs and cell culture media. At each time point, cells were harvested, washed with PBS and fixed with 70% ethanol solution overnight at −20 °C. After fixation, the cells were washed with PBS followed by centrifugation (2×). The cells were subsequently stained with 400 μL of FxCycle^TM^PI/RNase staining solution and incubated for 30 min at room temperature in the dark before flow cytometry analysis. Data were collected using a BD FACSVerse flow cytometer (BD Biosciences) and analyzed using FlowJo software version 10.4.0 (Tree Star Inc., Ashland, OR, USA). The results represent the average of three independent experiments.

##### Caspase 3/7 Activity Assay

Caspase activity was evaluated using the CellEvent^TM^ Caspase-3/7 Green Flow Cytometry Assay Kit by modifying a published protocol [33]. LNCaP and PC-3 cells (2.5 × 10^5^ cells/mL) were seeded in 6-well plates. After a 24-h incubation period, cells were treated with brusatol solution (40 nM), docetaxel solution (60 nM), combination drug solution (40 nM brusatol + 60 nM docetaxel), and nanoparticle suspension containing the same drug concentration as the combination drug solution for 72 h. Control cells were incubated with cell culture media. After 72 h, cells were harvested, and the cell suspension was made up to a 1 mL volume in flow cytometry tubes. CellEvent^TM^ Caspase-3/7 Green Detection Reagent (1 µL) was added to each sample tube and incubated for 25 min at 37 °C. SYTOX^®^AADvanced™ dead cell stain solution (1 mM; 1 μL) was added to the samples and incubated at 37 °C for 5 min. Samples were analyzed using a flow cytometer equipped with a 488 nm laser and 530/30- and 690/50 BP filters for fluorescence excitation following the manufacturer’s protocol.

##### Immunoblot Analysis

This was carried out using a modified published method [32]. LNCaP (2.5 × 10^5^ cells/mL) were cultured in 6-well plates and incubated overnight. The cells were treated with brusatol (40 nM), docetaxel (60 nM), combination drug solution (40 nM brusatol + 60 nM docetaxel), and nanoparticle suspension containing the same concentration of drugs as the combination drug solution for 24 h and 72 h. Control cells were incubated with cell culture media. After the treatment, the cells were harvested and lysed in 1 × SDS sample buffer, Halt^TM^ Protease and Phosphatase Inhibitor Cocktail, and 3% β-mercaptoethanol. Equal amounts of lysate (30 μL) were loaded into wells of 4–20% Tris-Glycine gels and proteins were separated by polyacrylamide gel electrophoresis. The separated proteins were transferred to a nitrocellulose membrane followed by blocking for 1 h at room temperature with constant shaking using a 1:5 ratio of milk in PBS containing 0.02% Tween 20. After blocking, the membranes were incubated overnight at 4 °C in a solution consisting of primary antibody in a 1:10 ratio of milk in PBS containing 0.02% Tween 20. Following overnight incubation, the membranes were washed for 5 min (3×) with a 1:20 dilution of milk in PBS solution containing 0.02% Tween 20, followed by incubation with secondary antibodies for 30 min under continuous shaking in a 1:10 ratio of milk in PBS containing 0.02% Tween 20 at room temperature. The membranes were washed for 5 min (3×) with a 1:20 dilution of milk in PBS solution containing 0.02% Tween 20. The membrane was exposed to a 1:1 solution of substrate luminal/enhancer solution and image captured using a LI-COR Odyssey Fc Imaging System (Lincoln, NE, USA).

### 2.5. Statistical Analysis

The results of the assays are expressed as the mean value ± standard deviations of individual experiments. Comparisons of means were conducted using Welch’s one-way ANOVA (GraphPad Prism-Version 6.0 for windows). *p* values ≤ 0.05 were considered significant. IC_50_ values were calculated using an online tool: “Quest Graph™ IC_50_ Calculator.” AAT Bioquest, Inc. (https://www.aatbio.com/tools/ic50-calculator, accessed on 21 January 2023) [34].

## 3. Results and Discussion

Most chemotherapeutic agents are proliferation dependent in their mechanism of action and are active against rapidly dividing cells. In addition, prostate cancer is a heterogeneous disease and the drugs currently in use do not kill all populations of tumor cells leading to relapse and regeneration of the tumor. Resistance to docetaxel is another great challenge with chemotherapy of prostate cancer. To overcome these challenges and improve therapeutic efficacy, approaches and treatment options that synergize with docetaxel to overcome these challenges are required. Brusatol is a protein synthesis inhibitor with specific inhibitory activity on Nrf2. Because it inhibits protein synthesis, it has the potential to kill all subpopulations of the tumor. As a result of its mechanism of action, and other challenges to its clinical use, targeting or site-specific delivery of brusatol to tumors is essential. In addition, the effect of brusatol on Nrf2 is short-lived. We hypothesize that sustained release of brusatol from a carrier or drug delivery system over time can ensure a prolonged effect on Nrf2 similar to what was obtained with repeated dosing. In addition to its cytotoxic effects as explained above, brusatol can also prevent the development of drug resistance and reverse the resistance to docetaxel as a result of its inhibition of Nrf2. Furthermore, the development of combination docetaxel- and brusatol-loaded nanoparticles and its selective targeting to the tumor microenvironment can augment radiotherapy via tilting the ROS balance to ensure very high levels of ROS in the tumor microenvironment, leading to cell death.

Thus, this work is novel, and the nanoparticle platform is essential: (a) to impact the biodistribution of encapsulated drugs and prevent or reduce toxicity to healthy cells (since the drugs will be released in the tumor environment as a result of the EPR effect and other active transcytosis mechanisms), (b) for sustained release and thereby reverse the short-lived effect of brusatol on Nrf2, and (c) to increase nanoparticle accumulation and drug concentration in the tumor, and thereby improve therapeutic efficacy.

Nanoparticle drug delivery systems have the potential to minimize toxicity and improve safety and efficacy in cancer therapy [35]. Several features of nanoparticles that are important for clinical use and for tumor targeting via the enhanced permeability and retention (EPR) effect have recently been discussed [26]. A significant challenge to the clinical translation of nanoparticle-based drug delivery is the rapid clearance of nanoparticles by the cells of the reticuloendothelial system (RES), which can limit their circulation time in the bloodstream and thus, reduce their efficacy. The size, shape, charge, and the nature of the nanoparticle surface are some of the properties that impact rapid nanoparticle clearance [36,37]. To minimize nanoparticle clearance and facilitate improved passive targeting to tumors, researchers have developed “stealth” nanoparticles. Stealth nanoparticles are nanoparticles surface-modified with hydrophilic, neutral polymers such as polyethylene glycol (PEG), to enhance circulation time by facilitating a reduction in the interaction of nanoparticles with the immune system. Additionally, particle size has been reported to be a major parameter that can be modified to improve the efficiency of nanoparticle accumulation and facilitate passive targeting to tumors. However, more recent data suggest that active transcytosis mechanisms may be more efficient for nanoparticle and macromolecule uptake into solid tumors when compared with passive targeting by the enhanced permeability and retention (EPR) effect [38,39]. Generally, research has shown that smaller nanoparticles are better able to avoid RES uptake and passively accumulate in tumors by the enhanced permeability and retention (EPR) effect [26,35,40]. Thus, our initial efforts were directed towards strategies and methods to identify formulation and process variables and their interactions that significantly impact the particle size of PLGA-PEG nanoparticles with the goal of particle size minimization to reduce uptake by the mononuclear phagocyte system and facilitate improved tumor localization and accumulation. To ensure robust and unbiased results, preparation and optimization of the nanoparticle formulation was carried out in two stages. Stage 1 included preliminary one-factor-at-a-time experiments to screen for factors that influenced nanoparticle size (Table 3). This was carried out to identify critical factors that impact nanoparticle size and to limit the number of factors in the statistical experimental design space. Stage 2 included full factorial statistical experimental design using the identified factors identified in stage 1 (Table 4).

### 3.1. Screening of Formulation and Process Variables Using the One-Factor-at-a-Time Approach

The emulsification-solvent evaporation technique was used for nanoparticle preparation. To minimize the size of nanoparticles, several formulation and process variables must be evaluated to determine their impact on particle size. Preliminary experiments were carried out using the one-factor-at-a-time approach, to screen for formulation and process variables that influence nanoparticle size. The eight factors evaluated include solvent composition of the organic phase, polymer concentration, organic to aqueous ratio, PVA (stabilizer) concentration, amplitude of sonication, on and off pulse duration, and sonication time. The factors evaluated and the particle sizes obtained by DLS are presented (Table 3).

To evaluate the effect of solvent composition on particle size, the ethyl acetate: DMF solvent mixture gave the smallest mean particle size. This is similar to earlier reports of the effects of different solvents on particle sizes of nanoparticles prepared using the nanoprecipitation method [41]. They suggested that better solvent diffusion and polymer dispersion into water is responsible for the relatively smaller sizes as a result of the greater miscibility of DMF in water compared to other solvents tested. This mechanism may also be applicable to nanoparticle fabrication using the emulsification-solvent evaporation method. For the effect of the organic solvent ratio on particle size, a solvent ratio of 1.6 mL of ethyl acetate to 0.4 mL of DMF gave the smallest mean particle size. At higher amounts of DMF in the organic solvent ratio, much larger particle sizes were obtained. When polymer concentrations were varied, the smallest mean particle size of 185.7 nm was obtained with the formulation using a polymer concentration of 75 mg/2 mL. Generally, with the increase in polymer concentration from 50 mg/2 mL to 100 mg/2 mL, there was not much difference in particle size. However, with a polymer concentration greater than 100 mg/2 mL, a considerable increase in the particle size was observed. It has been reported that, with an increase in polymer concentration, the viscosity of the organic phase is increased which decreases the efficiency of the sonication process, leading to bigger droplets being formed, resulting in increased particle size [3].

The effect of organic solvent phase to aqueous phase ratio was also evaluated and results indicated that 2 mL of organic phase to 12 mL of aqueous phase gave the smallest mean particle size. Larger particle sizes were obtained with an increase in the volume of the aqueous phase. These results may be due to the result of reduced sonication efficiency at larger formulation volumes leading to impaired droplet breakdown [42]. To evaluate the effect of stabilizer concentration, the smallest mean particle size was achieved with a 0.5% w/v PVA solution, while the largest sizes were observed with 2% PVA and 3% PVA. The increase in particle size at higher PVA concentrations can be attributed to the higher viscosity of the aqueous phase, which reduces the efficiency of the sonication process. On the other hand, the 0.25% w/v PVA solution may not provide sufficient stabilization of the formulation, resulting in larger particle sizes [42]. For the process variables, as the sonicator amplitude was increased, larger particle sizes were obtained at constant volume and polymer concentration. Additionally, the effect of the “on/off” pulse cycle on the sonication process was also evaluated. With 9 s on and 1 s off pulsing, the smallest mean particle size of 175.9 nm was obtained. Pulsing may enhance polymer processing by allowing the material to settle back under the probe after each burst. Because of this known mechanism, additional formulations (in triplicate) were prepared, which showed no difference in the particle size of formulations prepared using a pulse cycle of 9 s on and 1 s off compared to a pulse cycle of 8 s on and 2 s off; hence, the latter pulse cycle was selected. Further, a sonication time of 10 min gave the smallest mean particle size (Table 3). Generally, the nanoparticle sizes were impacted by process variables to the extent of formulation material processing.

While the ethyl acetate: DMF solvent mixture gave the smallest particle size of 202.8 nm, toxicity concerns with DMF, and the differences in the solubility profile of brusatol, docetaxel, and polymer favored the choice of ethyl acetate; the DMSO mixture for nanoparticle fabrication. Since this solvent composition gave larger particle sizes, the effect of acetone on the reduction of particle size was evaluated based on literature reports that the addition of acetone to the organic phase of nanoparticle formulations facilitates a reduction in particle size [43]. The authors reported that the addition of acetone alters the electrically charged surfaces of the PLGA-based nanoparticles leading to a decrease in the interfacial tension and other changes, ultimately facilitating a decrease in particle size. Thus, the effect of a two-solvent system (ethyl acetate: DMSO) versus a three-solvent system (ethyl acetate: DMSO: acetone) was evaluated. The average particle size obtained using the three-solvent system was 194.03 ± 3.93 (*n* = 3), which is smaller in comparison to the size obtained using the two-solvent system. Based on these data, which confirm the literature reports, and the fact that we could replace half the volume of the non-volatile DMSO in the initial formulation with acetone, the decision was taken to prepare nanoparticles using the three-solvent system.

### 3.2. Design of Experiments—Full Factorial Statistical Experimental Design

After obtaining preliminary results using the one-factor-at-a-time approach, the independent variables that were considered to have considerable effect on particle size and worthy of further evaluation (polymer concentration, organic: aqueous ratio, PVA concentration, and amplitude of sonication) were selected for statistical experimental design (Table 4). Other variables such as the sonication time, solvent composition, pulse cycle, and solvent ratio of the organic phase were kept constant. Factorial experimental designs, a DOE approach, allows for the simultaneous variation of all factors and analyzes the main effects and interactions between the independent or experimental variables and nanoparticle properties or response variables [29]. In addition, the model obtained from these studies can further be used for the optimization and prediction of nanoparticle size. For this study, the objective was to generate a model that could be used for the optimization of particle size with the goal of particle size minimization.

The results (Table 4) showed nanoparticle sizes ranging from 158.4 nm–230 nm. Analysis of variance was used to identify terms and the interactions that impacted particle size. To improve the model, insignificant terms were removed by backward elimination. The Analysis of Variance (ANOVA) output from Minitab^®^ for the dependent variable is presented in Table 5.

Based on the Analysis of Variance (ANOVA) data (Table 5), variables with *p* values < 0.05 were considered significant, indicating that they have a significant effect on particle size. Data showed that the model was significant (*p* = 0.000) and that the lack-of-fit was not significant (*p* = 0.466), suggesting that the model was well-fitted for the data and the linear model was acceptable for response prediction. The main effect that was found to be significant for average particle size was the organic to aqueous ratio (*p* = 0.000), and the two-way interaction between polymer concentration and organic to aqueous ratio was also significant (*p* = 0.03).

### 3.3. Regression Equation

Regression equations are mathematical equations that are used to predict the value of one variable based on the values of other variables in a dataset. With statistical analysis of the results, it is possible to determine values of regression coefficients that describe the direction of the relationship between an experimental or independent variable and the variable response or dependent or outcome variable [29].

The resulting polynomial (model) shown in Equation (1) below describes the relationship between experimental variables and the particle size:

Mean Particle Size (nm) = 27.7 + 1.8 Pol. Conc. (mg/2 mL) + 514 Org:Aqueous − 30.1 PVA Conc. (%) + 1.84 Ampl (%) − 9.17 Pol. Conc. (mg/2 mL) × Org:Aqueous + 183 Org:Aqueous × PVA Conc. (%)(1)

By using the regression equation above, it is possible to predict how a change in any of the dependent variables will affect particle size.

The optimization of particle size was conducted using the response optimizer feature based on the established optimized model. Table 6 displays the solutions generated by response optimization. This includes the values proposed for each factor to achieve particle size minimization and the composite desirability of the proposed solution (Supplementary information; Figure S1). The composite desirability is a statistical method used to evaluate the overall desirability of a formulation based on multiple criteria or responses. This value represents how well a particular formulation meets the desired specifications for each response or criterion. The composite desirability value ranges from 0 (undesirable) to 1 (perfectly desirable) [44]. Our data suggest that the formulation closely meets the desired specifications for each response, indicating that the optimization process was effective.

To check the validity of the model, the predicted optimized nanoparticle formulation solutions were experimentally prepared in triplicate and the mean particle sizes determined. Model verification was carried out by comparing the predicted particle sizes of the suggested optimized solutions with the mean particle size of the prepared nanoparticles. The results show that the model fits the data well (Table 6). Since the goal was particle size minimization, the predicted solution 1 was selected for drug-loading studies.

### 3.4. Nanoparticle Morphology, Particle Size, and Zeta Potential Characterization

**Figure 1:**
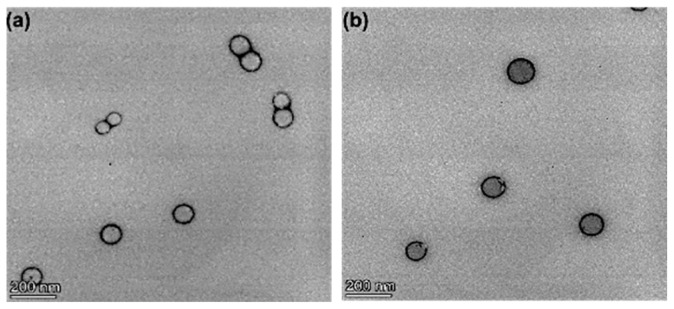
Typical TEM images of optimized formulation 1 (**a**) and optimized formulation 2 (**b**) showing the structure of the nanoparticles.

**Figure 2:**
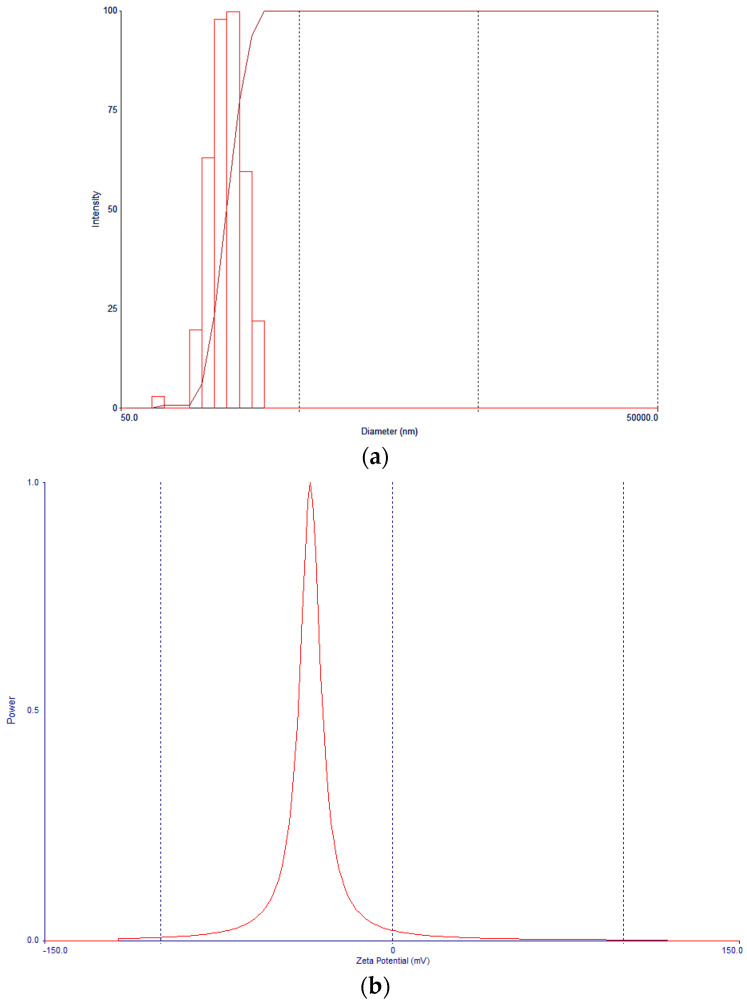
(**a**) Typical particle size and size distribution of nanoparticles, (**b**) Zeta potential of nanoparticles.

The morphological evaluation of negatively stained nanoparticles by transmission electron microscopy confirmed the formation of smooth spherical particles with a corona-core structure corresponding to the presence of a PEG corona overlaying the hydrophobic PLGA core (Figure 1). This TEM data show the potential for “stealth” capability in vivo. The mean particle sizes were determined by dynamic light scattering (DLS) (Figure 2a) and Figure 2b shows negative zeta potential. An average zeta potential value of −34.39 ± 0.48 mV was obtained for the optimized nanoparticle formulation. The zeta potential gives an effective electric charge on the nanoparticle surface [31], thus, the large negative value obtained is expected to provide sufficient interparticle repulsion thereby reducing the likelihood of aggregation in nanoparticle suspensions and therefore increased stability of the nanoparticle dispersions.

### 3.5. Drug-Loading Studies

The optimized formulation (solution 1) was used to prepare brusatol- and docetaxel-loaded nanoparticles. The drug content of the nanoparticle formulations prepared using different feed ratios of the individual drugs was determined by HPLC (Table 7). An increase in particle size of the drug-loaded nanoparticles was observed when compared to blank nanoparticles using the optimized formulation. This result may be attributed to encapsulation of the drugs in the PLGA core which leads to changes in the packing structure of the polymer during nanoparticle preparation [45]. Additionally, our data show that as the feed amount of docetaxel relative to brusatol increased, and the percent loading of docetaxel increased. This may be attributed to competition between the drugs for the limited space in the PLGA core of the nanoparticles [46]. Thus, the drug with the greater affinity for the hydrophobic PLGA core is expected to have improved loading within the nanoparticle core. One measure of the affinity of the drugs for interaction with the hydrophobic PLGA is the log *p* value of the drug. Generally, as the value of log P increases, the more lipophilic the drug is and a greater interaction between PLGA and the drug is expected. Thus, the improved loading of hydrophobic docetaxel (Log P—4.1) [47] over the less hydrophobic brusatol (Log P—1.82) [48] may be related to their affinities for the hydrophobic PLGA core. Furthermore, the data obtained show that increasing the amount of the drug to 30% of the polymer did not considerably improve brusatol loading, suggesting poorer loading efficiency at higher feed amounts of the drug.

For drug-loading studies, the goal was to use brusatol as an adjunct to docetaxel. In this way, a high percentage loading of docetaxel was desired with a reasonable percent loading of brusatol. This way, the amount of polymer injected would be minimal and at the same time, a sufficiently high amount of brusatol will be delivered. Thus, the optimized formulation with a feed ratio of 2.5:7.5 (docetaxel:brusatol) was selected for biological evaluations.

### 3.6. FT-IR Spectroscopy Evaluation

**Figure 3:**
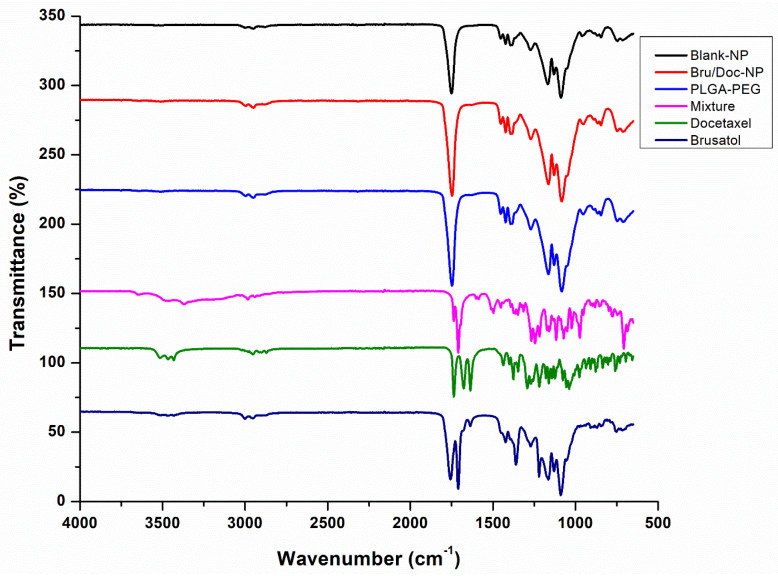
Overlay of FT-IR spectra of blank nanoparticles (black), brusatol- and docetaxel-loaded nanoparticles (red), pure brusatol (dark blue), pure docetaxel (green), PLGA-PEG polymer (purple), physical admixture (blue).

To evaluate potential interactions between the two drugs and components of the nanoparticle formulation and to qualitatively evaluate the efficiency of encapsulation of both drugs, FT-IR spectra were acquired for blank nanoparticles, brusatol- and docetaxel-loaded nanoparticles, pure brusatol, pure docetaxel, PLGA-PEG polymer, and physical admixture of polymer and drugs. The acquired spectra were overlaid (Figure 3). No drug peaks could be seen in the spectrum obtained for drug-loaded nanoparticles, while drug peaks are visible in the spectrum obtained for the admixture of polymer and drugs. In addition, the spectra obtained for the PLGA-PEG polymer, drug-loaded nanoparticles, and for blank nanoparticles are comparable and superimposable, suggesting that the drugs are completely encapsulated by the polymer.

### 3.7. Drug Release Studies

**Figure 4:**
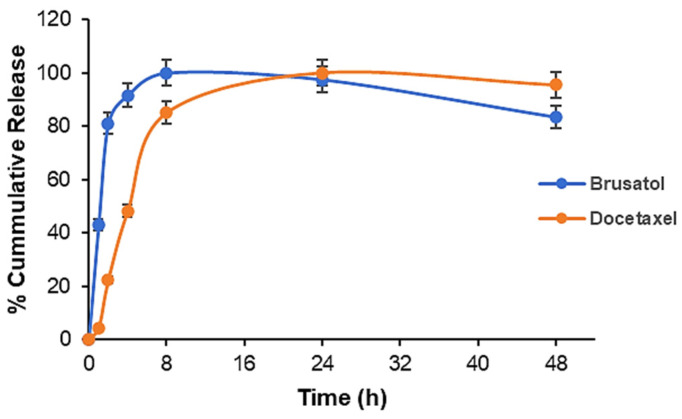
In vitro drug release profile of brusatol- and docetaxel-loaded nanoparticles in PBS at 37 ± 1 °C (*n* = 3; data represent mean ± SD).

The in vitro release isotherm of both drugs in PBS at pH 7.4 is presented in Figure 4. The data show that approximately 43% of brusatol was rapidly released during the first 1 h, whereas only about 4% of docetaxel was released within the same time frame. Release was completed for brusatol at 8 h, while docetaxel release was still ongoing at 24 h. The higher “burst release” observed with the brusatol release from nanoparticles compared to docetaxel could be due to several factors such as differences in solubility of the individual drugs, the interaction between the drugs and polymer, and percent loading of the drugs among others [49].

An important observation from a stability standpoint is that the encapsulation of both docetaxel and brusatol in the polymeric nanoparticle platform did not lead to any obvious degradation of the individual drugs. The retention time of either docetaxel or brusatol did not change when analyzed by HPLC in drug release studies.

### 3.8. Evaluation of Cytotoxicity of Pure Drugs and Determination of Combination Index

**Figure 5:**
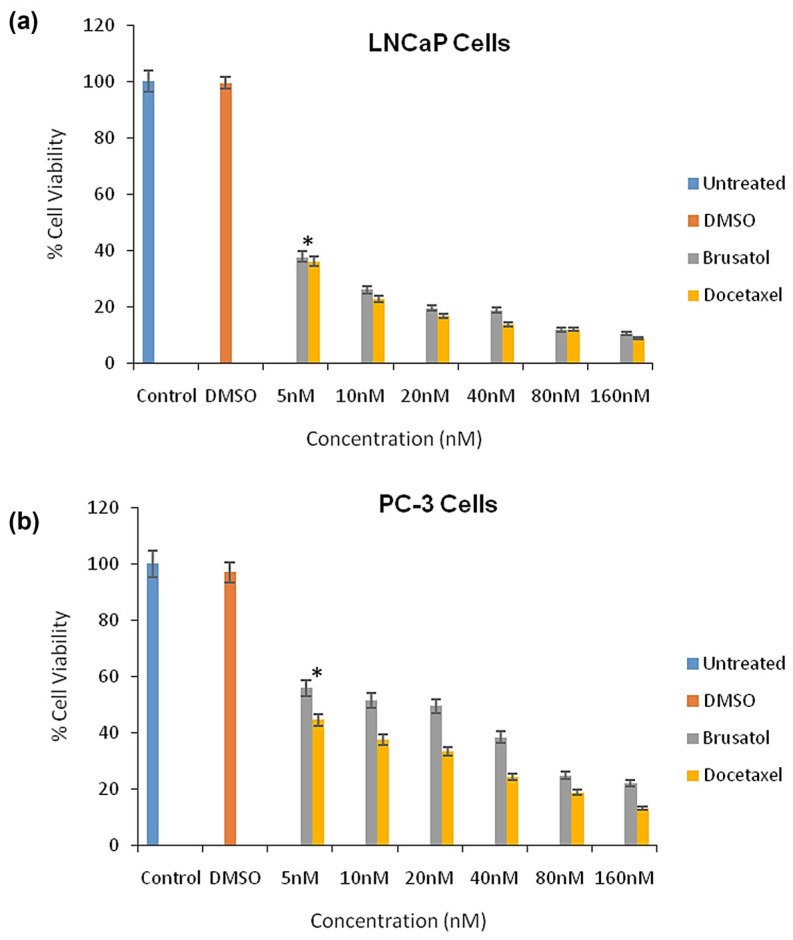
Percent cell viability of (**a**) LNCaP cells; (**b**) PC-3 cells treated with brusatol and docetaxel in vitro at 72 h (mean ± SD; *n* = 4). Control represents media only and DMSO represents 0.01% DMSO in media. Statistical analysis by two-way ANOVA and Paired *t*-test * *p* < 0.05.

The cytotoxicity of brusatol and docetaxel as single agents (Figure 5) and as a mixture (Supplementary information; Figure S2) of the single agents was evaluated using the XTT assay in LNCaP, and PC-3 cell lines at 72 h post treatment. As shown in Figure 5, brusatol and docetaxel inhibited the growth of LNCaP and PC-3 cells at 72 h post treatment in a concentration-dependent manner. The percent viability of LNCaP cells was lower than that of PC-3 cells when treated with similar concentrations, suggesting that LNCaP cells are more sensitive to the drugs. For the docetaxel-treated LNCaP and PC-3 cells, an estimated IC_50_ of 1.58 nM and 3.04 nM, respectively, was determined (Supplementary information; Figure S3). This result corresponds with what has been previously reported in the literature [50]. Similarly, with brusatol-treated LNCaP and PC-3 cells, an IC_50_ of 4.15 nM and 15.3 nM, respectively, was obtained (Figure S3). These IC_50_ values corroborate the observation that LNCaP cells are more sensitive to the drugs compared to PC-3 cells.

The combination index (CI), which is a quantitative measure of drug combination effects, can be used to determine the effects of the combination of brusatol and docetaxel on cytotoxicity in LNCaP and PC-3 cells [51,52]. The Chou-Talalay combination index provides a quantifiable index of interaction (synergism (CI < 1), additive effect (CI = 1), and antagonism (CI > 1) in drug combinations [53]. For this work, to quantify the cytotoxic effect of the interaction of brusatol and docetaxel in human prostate cancer cell lines and to justify the combination of both drugs in a nanoparticle platform, LNCaP and PC-3 cells were exposed to a 50:50 ratio of brusatol and docetaxel at concentrations (Supplementary Information; Figure S2) over the same range as presented for single drugs over a 72-h period. The CI was determined using the Chou-Talalay CI tool (CompuSyn software, version 1.0).

As shown in Table 8, the CI values of different combinations of drug concentration ranged from 0.33061 to 0.95830 for LNCaP cells, indicating synergism (Supplementary Information; Figure S4a). A concentration-dependent effect on CI values was observed. As the concentration of combination drug solution increased, the CI value increased with the lower concentrations, showing stronger synergism (Table 6). Similarly, the CI values of drug combinations on PC-3 cells ranged from 0.10535 to 0.99662, indicating synergism (Supplementary information; Figure S4b). A similar concentration-dependent effect on CI values was also observed. An important observation is that strong synergism was observed at a total dose at or below the IC_50_ of each drug in both cell lines. Thus, based on the CI data showing synergism, the combination of brusatol and docetaxel in a nanoparticle platform for combination nanotherapeutics is justified and may facilitate significantly improved anti-tumor activity and cytotoxicity effects in human prostate cancer.

### 3.9. Cytotoxicity Evaluations of Brusatol- and Docetaxel-Loaded Nanoparticle Formulations

#### 3.9.1. Viable Cell Count and In Vitro Cytotoxicity

**Figure 6:**
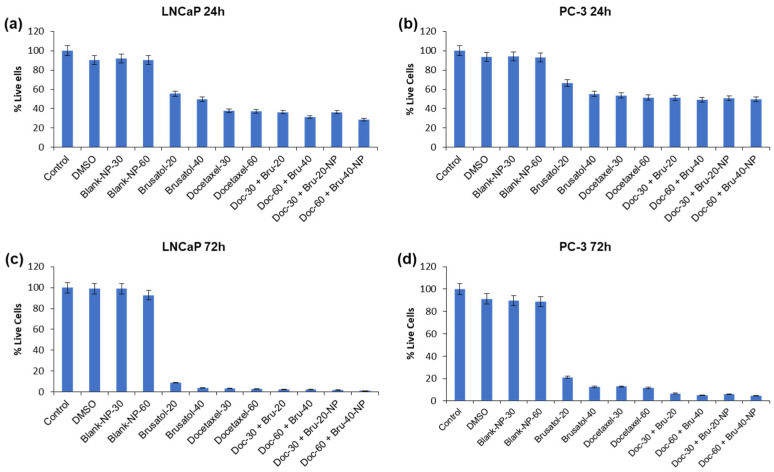
Evaluation of cytotoxicity of docetaxel- and brusatol-loaded nanoparticles and controls at different concentrations by automated viable cell count. (**a**) LNCaP cells (24 h), (**b**) PC-3 cells (24 h), (**c**) LNCaP cells (72 h), (**d**) PC-3 cells (72 h). Data are mean ± SD (*n* = 3).

**Figure 7:**
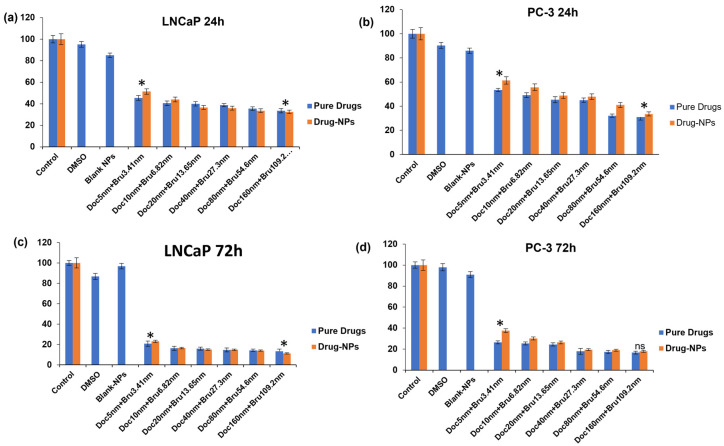
In vitro evaluation of cytotoxicity of combination drug solution, and combination drug-loaded nanoparticles containing the same concentration of drugs as the solution, and other controls at different concentrations of drugs using the MTS assay. (**a**) LNCaP cells (24 h), (**b**) PC-3 cells (24 h), (**c**) LNCaP cells (72 h), (**d**) PC-3 cells (72 h). Data are mean ± SD (*n* = 3). Statistical analysis by 2-way ANOVA and Paired *t*-test * *p* < 0.05.

To evaluate the cytotoxicity of the optimized brusatol- and docetaxel-loaded nanoparticle formulation, cells seeded in wells treated with different concentrations of drug-loaded nanoparticles and other controls were counted to determine the percentage of live cells relative to cells in untreated control wells. Figure 6 and Figure 7 show the percent viability of LNCaP and PC-3 cells after treatment with different concentrations of test agents at 24 h and 72 h. In general, the nanoparticle formulation, like the combination drug solution, showed time-dependent toxicity against LNCaP and PC-3 cells with more cell death observed at 72 h compared to 24 h in both cell lines. In addition, blank nanoparticles did not show toxicity to cells, confirming that the toxicity observed is due to the encapsulated drugs.

#### 3.9.2. Cell Cycle Analysis

Docetaxel and brusatol are two chemical compounds that have been extensively studied for their anti-cancer properties. These drugs work by targeting different phases of the cell cycle, with docetaxel primarily inducing arrest in the G2/M phase [50], and brusatol arresting cells in the G1 phase of the cell cycle [54,55].

**Figure 8:**
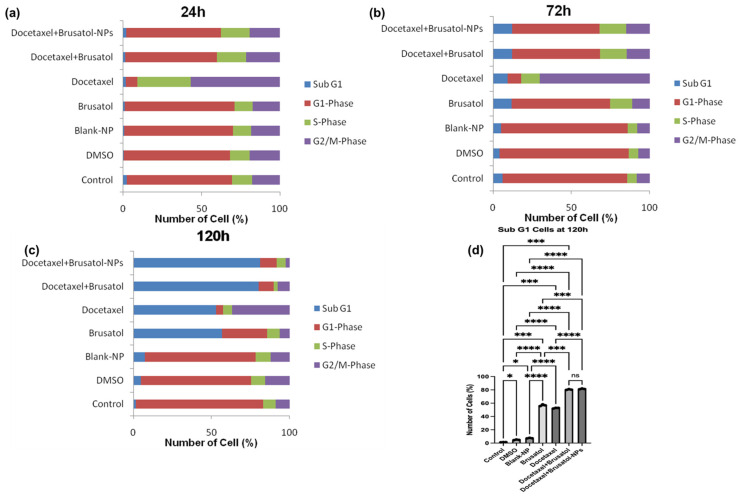
Effect of DMSO solution, blank-NPs, brusatol-40 nM, docetacel-60 nM, docetacel-60 nM + brusatol-40 nM solution, and docetacel-60 nM + brusatol-40 nM nanoparticles on cell cycle distribution of LNCaP cells post treatment (**a**) 24 h, (**b**) 72 h, (**c**) 120 h, (**d**) showing the population of cells in subG1-phase at 120 h post-treatment (*n* = 3). Statistical analysis by Brown-Forsythe ANOVA with Welch’s ANOVA test * *p* ≤ 0.05, *** *p* ≤ 0.001, **** *p* ≤ 0.0001. ns means “not significant”.

**Figure 9:**
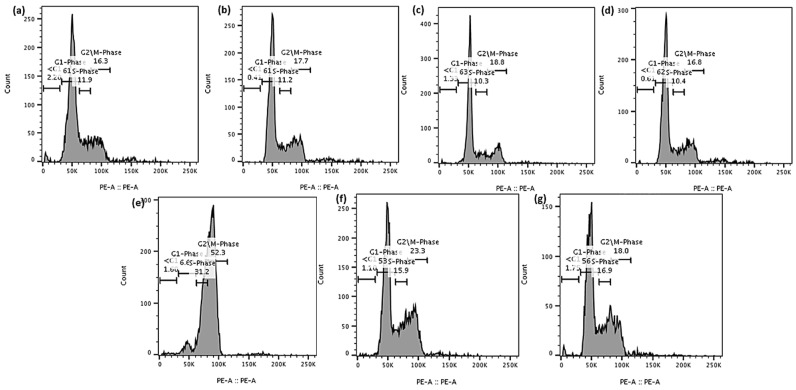
Representative flow cytometry histograms of cell cycle analysis of LNCaP cells treated with (**a**) Control (**b**) DMSO, (**c**) blank-NPs, (**d**) brusatol-40 nM, (**e**) docetaxel-60 nM, (**f**) docetaxel-60 nM + brusatol-40 nM solution, (**g**) docetaxel-60 nM + brusatol-40 nM nanoparticles and incubated for 24 h. Table of average values is presented in the supporting information (Table S1).

In this study, PC-3 and LNCaP cells treated with brusatol- and docetaxel-loaded NPs, single drugs, and other controls were evaluated by flow cytometry (Supplementary Information; Table S1) after staining with propidium iodide (PI) in RNase solution. PI is a fluorescent dye that binds DNA and only enters dead or dying cells with damaged or leaky membranes. Because of this characteristic, it is used as a marker for apoptotic and necrotic cells [56]. As shown in Figure 8 and Figure 9, treatment of LNCaP cells with brusatol as a single agent at about 40 nM resulted in 66.6% of the cells being arrested in the G1-phase of the cell cycle at 24 h. This is supported by previous studies where antitumor and anticancer activities of brusatol have been shown to induce cell cycle arrest in the G1-phase before promoting tumor cell death [54,55]. At 72 h (Supplementary Information; Figure S7) post treatment, about 60.7% of LNCaP cells show G1-phase arrest with 11.2% of cells observed in the sub-G1 phase, indicative of cell death. Further, analysis of the cell cycle at 120 h (Supplementary information; Figure S8) shows that brusatol treatment caused considerable cell death due to a large increase in the subG1 phase cell population (about 54.2%). This cell death by brusatol has been reported [54,55,57].

Treatment of LNCaP cells with docetaxel as a single agent at about 60 nM for 24 h resulted in about 52.3% of the cells arrested in the G2/M phase and 66.9% arrested in the G2/M phase at 72 h. At 120 h post treatment, this percentage decreased to about 35.6% of cells arrested in the G2/M phase with a concomitant increase in the percentage of cells in the subG1 phase (51%). This finding is consistent with earlier studies, which found that docetaxel treatment resulted in a substantial increase in the percentage of cells arrested in the G2/M phase before inducing cell death in LNCaP and PC-3 cells as a result of apoptosis [50,58,59].

The treatment of LNCaP cells with a solution containing a mixture of docetaxel (60 nM) and brusatol (40 nM) resulted in about 54.8% of the cells arrested in the G1-phase of the cell cycle at 24 h and 54.1% at 72 h. Even though about 54% of G1-phase arrest was observed at both time periods, time-dependent cell death was observed, with 1.27% of the cells at the subG1 phase at 24 h, and 11.7% of the cells in the subG1 phase at 72 h. In addition, about 20.2% and 14.2% of the cells were arrested at 24 h and 72 h, respectively, in the G2/M phase of the cell cycle. Furthermore, LNCaP cells treated with the optimized docetaxel- and brusatol-loaded nanoparticle formulation containing the same concentrations of drug as the combination drug solution displayed similar cell cycle arrest characteristics. At 120 h post treatment with both solution and nanoparticle formulation (Figure 8d), the number of LNCaP cells in the subG1 phase, an indication of cell death, increased to approximately 78.6% and 79.7%, respectively.

**Figure 10:**
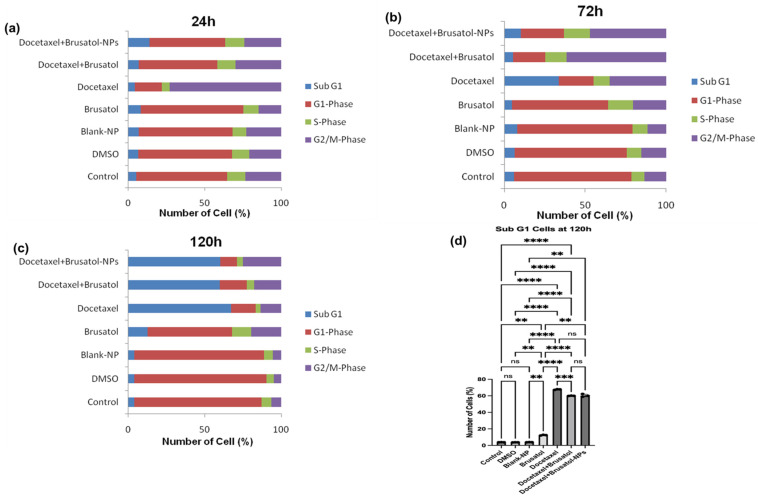
Effect of DMSO solution, blank-NPs-60 nM, brusatol-40 nM, docetaxel-60 nM, docetaxel-60 nM + brusatol-40 nM solution, and docetaxel-60 nM + brusatol-40 nM nanoparticles on cell cycle distribution of PC-3 cells post treatment (**a**) 24 h, (**b**) 72 h, (**c**) 120 h, (**d**) the population of cells in subG1-phase at 120 h post-treatment (*n* = 3). Statistical analysis by Brown-Forsythe ANOVA with Welch’s ANOVA test ** *p* ≤ 0.01, *** *p* ≤ 0.001, **** *p* ≤ 0.0001. ns means “not significant”.

**Figure 11:**
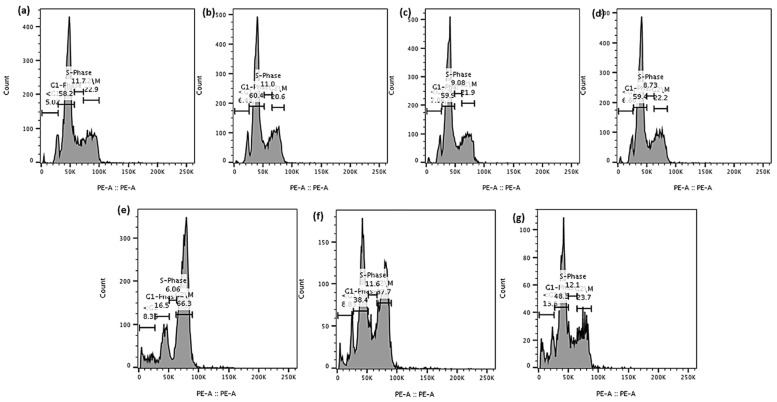
Representative flow cytometry histograms of cell cycle analysis of PC-3 cells treated with (**a**) control, (**b**) DMSO solution, (**c**) blank-NPs, (**d**) brusatol-40 nM, (**e**) docetacel-60 nM, (**f**) docetacel-60 nM + brusatol-40 nM solution, (**g**) docetacel-60 nM + brusatol-40 nM nanoparticles and incubated for 24 h. Table of average values is presented in the supporting information (Table S1).

As shown in Figure 10 and Figure 11, the treatment of PC-3 cells with brusatol as a single agent at the same concentrations used for LNCaP cells resulted in about 66.1% of the cells being arrested in the G1-phase of the cell cycle at 24 h and a 57% G1-phase arrest at 72 h. At 120 h, 11.7% of cells were in the subG1 phase. A comparison of the percentage of cells in the subG1 phase at 120 h showed a considerable difference between LNCaP cell response to brusatol and PC-3 cell response, confirming that PC-3 cells are more resistant to brusatol.

Similarly, the treatment of PC-3 cells with docetaxel as a single agent for 24 h resulted in 70.6% of the cells arrested in the G2/M phase while at 72 h (Supplementary Information; Figure S9), 32.2% of the cells were arrested in the G2/M phase. It was also observed that at 72 h, the number of cells in the subG1 phase increased to 31%, an indication of increased DNA fragmentation and cell death. With the increase in time to 120 h post treatment, 66.3% of the cells were in the subG1 phase (Supplementary Information; Figure S10). Thus, our data show time-dependent cell death and that PC-3 cells are more sensitive to docetaxel compared to brusatol.

The treatment of PC-3 cells with a solution containing a mixture of docetaxel (60 nM) and brusatol (40 nM) for 24 h resulted in about 50.5% of cells arrested in the G1-phase and 29.4% arrested in the G2/M phase (Supplementary Information; Table S1). At 72 h post treatment, there was a substantial percentage drop to 17.8% of the cells arrested in the G1-phase compared to 24 h, while the percentage of cells in the G2/M phase increased to about 55.6% compared to the percentage of cells arrested in the G2/M phase at 24 h (Supplementary Information; Figure S9). At 120 h post treatment, an increase in the percentage of cells in the subG1 phase considerably increased to about 58.1% when compared to 6.74% and 4.84% obtained at 24 h and 72 h, respectively (Supplementary Information; Figure S10). For the nanoparticle formulation containing a combination of both drugs at the same concentrations as in the solution, a similar trend as for the mixture of the two drugs in solution was obtained.

In this study, cell cycle analysis data showed cell cycle arrest in both the G1-phase and G2/M phase in LNCaP and PC-3 cells with subsequent progression to cell death. In all cases, the data showed cell cycle arrest corresponding to the individual mechanisms of the component brusatol and docetaxel in the dual drug-loaded nanoparticles. Thus, this biphasic cell cycle arrest profile is expected to prevent the proliferation of cancer cells more efficiently and may contribute to the synergy observed with the cytotoxic agents.

#### 3.9.3. Caspase-3/7 Activity

**Figure 12:**
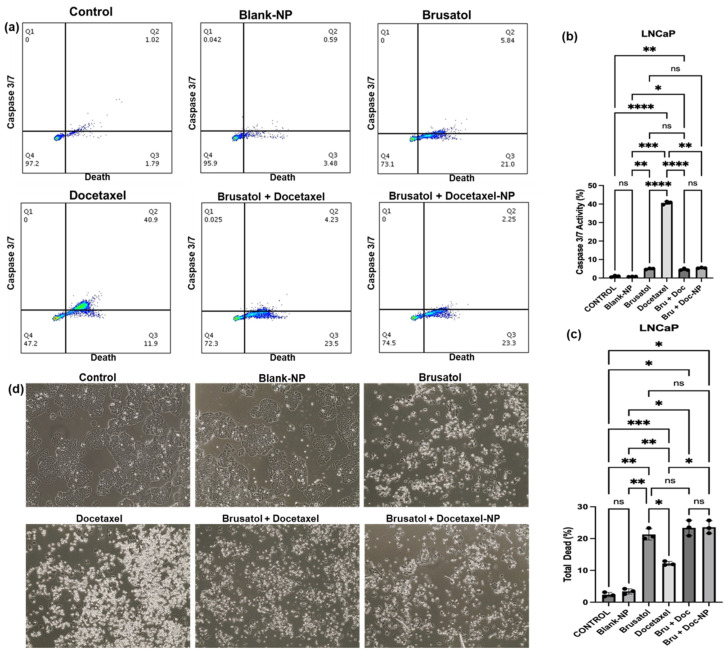
Caspase 3/7 activity in LNCaP cells treated with blank-NPs, brusatol-40 nM, docetaxel-60 nM, docetaxel-60 nM + brusatol-40 nM solution, and docetaxel-60 nM + brusatol-40 nM nanoparticles and incubated for 72 h. (**a**) Dot plot of caspase 3/7 activity, (**b**) Percentage of caspase 3/7 activity, (**c**) Percentage of total cell death, (**d**) Phase contrast images of treated cells; (×40 Magnification). Statistical analysis by Brown-Forsythe ANOVA with Welch’s ANOVA test * *p* ≤ 0.05, ** *p* ≤ 0.01, *** *p* ≤ 0.001, **** *p* ≤ 0.0001. ns means “not significant”.

Caspases are a family of proteases that play a crucial role in programmed cell death or apoptosis [60,61]. In particular, caspases are considered the executioners of the apoptotic pathway, and their activation leads to the cleavage of many cellular proteins, ultimately resulting in cell death [62]. Figure 12 depicts the level of caspase 3/7 activity and total cell death in LNCaP cells after treatment with the nanoparticle formulation containing the same concentration of drugs as in the combination drug solution (40 nM brusatol + 60 nM docetaxel), and controls, i.e., brusatol solution (40 nM), docetaxel solution (60 nM), and combination drug solution (40 nM brusatol + 60 nM docetaxel) for 72 h.

As shown in Figure 12b, treatment of LNCaP cells with 60 nM docetaxel increased caspase 3/7 activity to about 40.6% when compared to caspase 3/7 activity in cells treated with brusatol (4.7%), combination drug solution (4.6%), and optimized nanoparticle formulation (5.4%). The significant increase in the level of caspase 3/7 activity in LNCaP cells treated with docetaxel is supported by the literature, which shows that docetaxel causes tumor cell death by inducing caspase-dependent apoptosis and mitotic destruction after inhibiting microtubule depolymerization [63,64].

The percentage of total LNCaP cell death obtained after treatment with the different agents for 72 h is presented in Figure 12c. The percentages of death in cells treated with brusatol (21.3%), combination drug solution (23.4%), and nanoparticle loaded with equivalent amounts of pure drugs as combination drug solution (23.6%) were significantly higher compared to the percentage of cell death observed in cells treated with pure docetaxel (12.2%). In this experiment, even though lower caspase 3/7 activity was observed, the percentage of total dead cells are higher in brusatol-containing treatments compared to the docetaxel-treated group. This suggests that brusatol and brusatol-containing treatments are inducing cell death through alternative mechanisms other than the caspase 3/7 apoptosis pathway in LNCaP cells. It is worth noting that other mechanisms such as necrosis, autophagy, and ferroptosis that are triggered by chemotherapy may lead to cell death [65,66,67].

**Figure 13:**
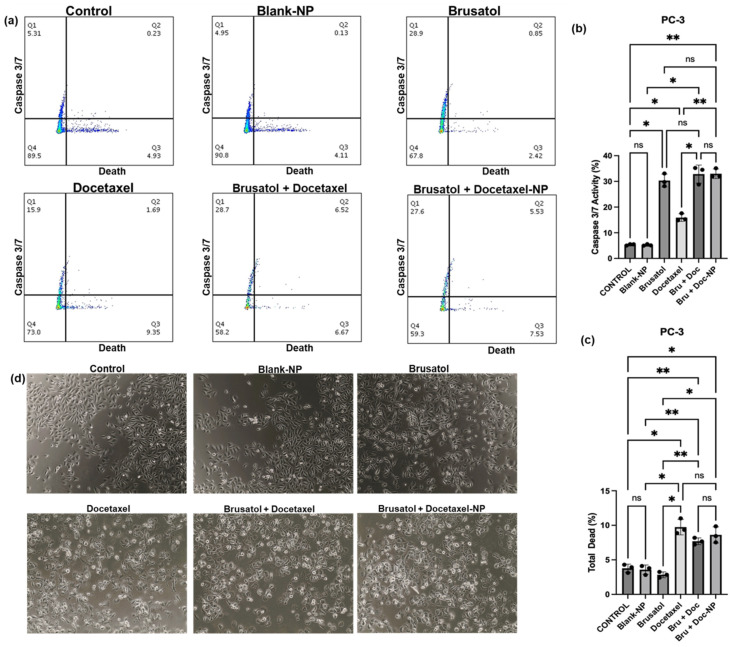
Caspase 3/7 activity in PC-3 cells treated with blank-NPs, brusatol-40 nM, docetaxel-60 nM, docetaxel-60 nM + brusatol-40 nM solution, and docetaxel-60 nM + brusatol-40 nM nanoparticles and incubated for 72 h. (**a**) Dot plot of caspase 3/7 activity, (**b**) Percentage of caspase 3/7 activity, (**c**) Percentage of total death, (**d**) Phase contrast images of treated cells; (×40 Magnification). Statistical analysis by Brown-Forsythe ANOVA with Welch’s ANOVA test * *p* ≤ 0.05, ** *p* ≤ 0.01. ns means “not significant”.

As shown in Figure 13, treatment of PC-3 cells with the nanoparticle formulation containing the same concentration of drugs as combination drug solution (40 nM brusatol + 60 nM docetaxel), brusatol (40 nM), and combination drug solution (40 nM brusatol + 60 nM docetaxel) for 72 h showed 33%, 30.3%, and 32.8% caspase 3/7 activity, respectively (Figure 13b), while PC-3 cells treated with docetaxel (60 nM) showed a lower caspase 3/7 activity of 15.9%. Thus, lower caspase activity was observed with docetaxel-treated PC-3 cells compared with docetaxel-treated LNCaP cells, where docetaxel treatment significantly increased caspase 3/7 activity.

With respect to total cell death, as shown in Figure 13c, the total cell death observed in PC-3 cells treated with brusatol (40 nM) was shown to be 2.84%, while the cells treated with docetaxel (60 nM), the combination drug solution (40 nM brusatol + 60 nM docetaxel), and nanoparticle formulations containing the same concentration of drugs as the combination drug solution showed no significant difference in total death at 9.74%, 7.7%, and 8.6%, respectively (*p* > 0.05). Thus, in PC-3 cells, our data show that total cell death is greatest in docetaxel-containing treatments (Figure 13c), unlike LNCaP cells where the total cell death is greatest in brusatol-containing treatments. These interesting results may contribute to differences in sensitivity to drug treatments between the two cell lines. Additionally, in the PC-3 cell line, the cell death observed is not limited to caspase activation alone since docetaxel treatment showed the least caspase 3/7 activation (Figure 13b). Furthermore, treatment with brusatol (40 nM) led to an increase in caspase 3/7 activity but showed the least cell death among the treatment groups, suggesting that the cell death observed is not mainly mediated by caspases.

Put together, the results obtained show that LNCaP cells have greater sensitivity to brusatol compared to PC-3, as earlier suggested by percentage viability data (Figure 5). Furthermore, phase contrast microscopy images of LNCaP cells (Figure 12d) and PC-3 cells (Figure 13d) treated with the different treatment groups and controls confirm morphological changes characteristic of cell death.

#### 3.9.4. Immunoblotting Assay

**Figure 14:**
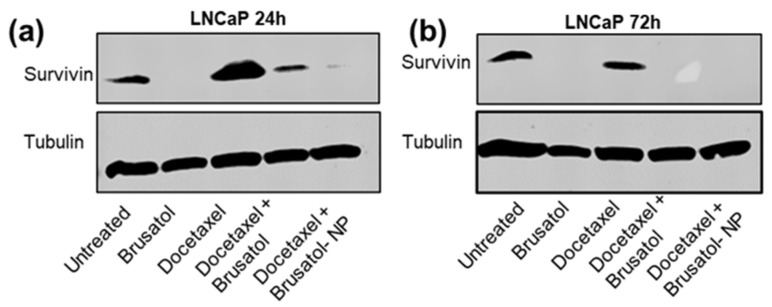
Immunoblotting assay: Western blot analysis for survivin to evaluate the effect of the different drug treatments on survivin expression using tubulin as control. (**a**) LNCaP cells at 24 h, (**b**) LNCaP cells at 72 h, after treatment with brusatol (40 nM), docetaxel (60 nM), combination drug solution (40 nM brusatol + 60 nM docetaxel), and nanoparticle formulations containing the same concentration of drugs as in the combination drug solution.

To unravel the mechanisms by which brusatol solution, docetaxel solution, combination drug solution, and nanoparticle formulations containing the same concentrations of drugs as the combination drug solution induce cell death in LNCaP cells, Western blots to detect the expression of survivin were carried out (Figure 14). Survivin is a member of the inhibitor of apoptosis (IAP) family of proteins that has been implicated in promoting cancer cell survival and resistance to chemotherapy [68,69,70]. It has been reported that the expression of survivin is upregulated in various cancer cell lines, including LNCaP cells, in response to treatment with chemotherapeutic agents such as docetaxel [71,72,73,74]. This upregulation of survivin is thought to be a survival mechanism that enables cancer cells to evade apoptosis and continue to proliferate [71,72,73,74]. Other groups have reported that the anti-apoptotic activity of survivin is dependent on mitotic phosphorylation to keep it stable [70]. The downregulation of survivin expression has been associated with the spontaneous apoptosis of cancer cells [70]. Additionally, high levels of survivin expression have been shown to correlate with poor prognosis in cancer patients [75,76], and the overexpression has been associated with chemoresistance [68,69]. Thus, the downregulation or inhibition of survivin may sensitize prostate cancer cells to chemotherapy-induced apoptosis and ultimately enhance the efficacy of chemotherapy.

Our results show that LNCaP cells expressed high levels of survivin after treatment with docetaxel for 24 h (Figure 14a). It has been reported that survivin is expressed during the G2/M phase of the cell cycle [76]. Thus, with G2/M arrest following docetaxel treatment, most of the cells are in the G2/M phase leading to the expression of high levels of survivin. Interestingly, survivin expression was not observed in LNCaP cells after treatment with brusatol for 24 h. This observation is in agreement with the mechanism of brusatol as a protein synthesis inhibitor [77,78].

For the 72-h studies, the downregulated expression of survivin was observed in LNCaP cells after treatment with docetaxel for 72 h compared to 24-h data. This downregulation in survivin levels could be correlated with the percentage of cells in the G2/M phase based on the cell cycle assay data discussed above when 24-h and 72-h data are compared. This time-dependent reduction in survivin levels when compared with the observed overexpression at 24 h may be one of the mechanisms responsible for the time-dependent increase in docetaxel cytotoxicity at longer treatment times. Additionally, the inhibition of survivin protein expression was observed at 72 h in LNCaP cells treated with brusatol (Figure 14b). Thus, no time-dependent difference in expression was observed in LNCaP cells treated with brusatol. 

When comparing survivin expression in cells treated with combination therapy, some interesting differences were noted. Generally, while the downregulation of survivin expression was observed in LNCaP cells at 24 h, no expression was observed at 72 h for the combination treatments. We postulate that this observation could be explained in part by the inhibitory effect of brusatol on the time-dependent expression of survivin facilitated by docetaxel at the different time points in LNCaP cells. At 24 h, the effect of the brusatol inhibition of survivin protein expression could not overcome the overexpression facilitated by docetaxel, hence only downregulated survivin expression was observed. This docetaxel-facilitated survivin overexpression was weaker at the 72-h time point, thus brusatol inhibition of survivin was effective, therefore, no survivin expression was observed. This interplay of inhibition, facilitated by brusatol, versus the overexpression facilitated by docetaxel at different time periods may be responsible for the observed differences in response to treatments. These results could be a significant finding, as it suggests that the nanoparticle formulation may be more effective in killing cancer cells. 

## 4. Conclusions

We successfully fabricated and characterized sub-200 nm combination docetaxel- and brusatol-loaded nanoparticles and evaluated their potential for prostate cancer therapy in vitro using PC-3 and LNCaP prostate cancer cells. Formulation and optimization data showed that the experimental design is robust and can be used to accurately predict particle size. Biological experiments using cell cultures revealed that the drug combination showed synergistic cytotoxic effects and revealed potential mechanisms responsible for the observed synergistic effects. The developed nanoparticles show promise for the treatment of prostate cancer and additional work is ongoing to target the nanoparticles to prostate specific membrane antigen overexpressed on cancer cells and to evaluate the therapeutic potential in animal models of prostate cancer.

## Figures and Tables

**Table 2:** Feed composition of drug-loaded nanoparticles (*n* = 3).

Formulation	Docetaxel (mg)	Brusatol (mg)	Drug:Polymer (%)
1	7	3	20%
2	6	4	20%
3	5	5	20%
4	4	6	20%
5	2.5	7.5	20%
6	3	7	20%
7	2	8	20%
8	1	9	20%
9	4.5	10.5	30%
10	3	12	30%

**Table 3:** Particle size data from initial screening experiments using the one-factor-at-a time method.

Formulation Factor	Variation	Size (nm)	Process Factor	Variation	Size (nm)
Solvent composition	Ethyl acetate:DMF	202.8	Pulse (on/off, sec)	No pulse	188.7
	Ethyl acetate:acetone	206.9		7:3	230.8
	Ethyl acetate:DMSO	212.8		8:2	178.1
	Ethyl acetate:ACN	207.6		9:1	175.9
Solvent ratio	1.2:0.8	371.6	Amplitude (%)	25	176.9
	1.4:0.6	391		30	196.1
	1.6:0.4	179.2		35	193.3
	1.8:0.2	200.4		40	203.8
Polymer conc.	20 mg/2 mL	207.1	Sonic. time (min)	2.5	199.3
	50 mg/2 mL	189.6		5	188.8
	75 mg/2 mL	185.7		7.5	186.8
	100 mg/2 mL	194.5		10	186.1
	150 mg/2 mL	355.1			
Organic:Aqueous	2:8	203			
	2:10	193.4			
	2:12	192.1			
	2:14	318.5			
	2:16	399.4			
PVA conc. (%)	0.25	186.9			
	0.5	182.1			
	1	184.5			
	2	195			
	3	195			

ACN—acetonitrile; DMF—Dimethylformamide; DMSO—Dimethyl sulfoxide.

**Table 4:** Full factorial statistical experimental design for the preparation of nanoparticles generated by Minitab^®^ with mean particle size and PDI of nanoparticle formulations.

Formulation	Polymer Conc. (mg/2 mL)	Organic:Aqu	PVA Conc. (%)	Amplitude (%)	Size (nm)	Polydispersity
1	25	2|8	1.5	30	225.2	0.149
2	50	2|8	0.5	30	212.5	0.048
3	25	2|8	1.5	26	230.4	0.134
4	50	2|8	1.5	26	201.7	0.104
5	50	2|8	1.5	30	206.7	0.049
6	37.5	2|10	1	28	195	0.081
7	25	2|8	0.5	30	220.5	0.064
8	50	2|8	1.5	30	212.9	0.058
9	50	2|12	0.5	30	203.3	0.067
10	25	2|12	1.5	26	186.7	0.128
11	25	2|8	1.5	26	223.8	0.105
12	37.5	2|10	1	28	201.8	0.088
13	50	2|12	1.5	26	185.6	0.032
14	37.5	2|10	1	28	190.6	0.116
15	50	2|8	0.5	26	197.8	0.089
16	25	2|12	1.5	30	177.3	0.102
17	50	2|12	0.5	30	181.6	0.059
18	25	2|8	1.5	30	210.5	0.098
19	25	2|12	1.5	26	158.4	0.08
20	25	2|12	0.5	30	173.3	0.114
21	37.5	2|10	1	28	184.9	0.046
22	50	2|12	1.5	30	181	0.085
23	25	2|12	0.5	26	173.7	0.042
24	25	2|8	0.5	26	203.3	0.079
25	50	2|8	0.5	30	192.3	0.089
26	25	2|12	1.5	30	170.4	0.093
27	37.5	2|10	1	28	175.1	0.147
28	25	2|8	0.5	30	186	0.075
29	25	2|12	0.5	30	167.7	0.119
30	25	2|8	0.5	26	181.9	0.079
31	50	2|12	0.5	26	173.1	0.069
32	50	2|12	1.5	26	172.7	0.077
33	50	2|8	1.5	26	184.1	0.106
34	50	2|12	1.5	30	176	0.061
35	25	2|12	0.5	26	171.7	0.105
36	50	2|8	0.5	26	183.8	0.138
37	50	2|12	0.5	26	159.6	0.071

**Table 5:** Analysis of variance table.

Analysis of Variance					
Source	DF	Adj SS	Adj MS	F-Value	*p*-Value
Model	6	9113.7	1518.94	10.91	0.000
Linear	4	7926.1	1981.54	14.23	0.000
Pol. Conc. (mg/2 mL)	1	62.7	62.72	0.45	0.507
Org:Aqueous	1	6903.1	6903.12	49.59	0.000
PVA Conc. (%)	1	528.1	528.12	3.79	0.061
Amp (%)	1	432.2	432.18	3.10	0.088
2-Way Interactions	2	1187.5	593.75	4.27	0.023
Pol. Conc. (mg/2 mL) * Org:Aqueous	1	723.9	723.90	5.20	0.030
Org:Aqueous * PVA Conc. (%)	1	463.6	463.60	3.33	0.078
Error	30	4176.4	139.21		
Curvature	1	4.4	4.36	0.03	0.863
Lack-of-Fit	9	1301.2	144.58	1.01	0.466
Pure Error	20	2870.8	143.54		
Total	36	13,290.1			

**Table 6:** Response optimization showing predicted and experimental mean particle size data (*n* = 3).

Solution	Pol. Conc. (mg/2 mL)	Org:Aqu	PVA Conc. (%)	Amp (%)	Predicted Particle Size (nm)	Composite Desirability	Experimental Mean Particle Size (nm)
1	25	0.167	0.5	26	168.333	0.868436	169.09
2	50	0.1670	0.5	26	175.046	0.77953	175.15

**Table 7:** Feed amount of brusatol and docetaxel and mean particle size and percent loading of each drug using the optimized nanoparticle formulation (*n* = 3).

Formulation	Doc (mg)	Bru (mg)	Size (nm)	Doc Loading (%)	Bru Loading (%)	Drug:Polymer (%)
1	7	3	201.87 ± 1.40	7.84 ± 0.17	0.87 ± 0.17	20%
2	6	4	195.70 ± 1.15	6.37 ± 0.11	0.79 ± 0.07	20%
3	5	5	198.27 ± 2.78	6.32 ± 0.11	1.14 ± 0.03	20%
4	4	6	182.57 ± 1.90	3.73 ± 0.25	0.82 ± 0.09	20%
5	3	7	194.97 ± 0.75	2.49 ± 0.13	0.79 ± 0.08	20%
6	2.5	7.5	184.80 ± 1.30	2.04 ± 0.04	0.89 ± 0.02	20%
7	2	8	188.80 ± 0.80	1.41 ± 0.17	0.99 ± 0.15	20%
8	1	9	193.83 ± 1.59	0.70 ± 0.06	1.48 ± 0.28	20%
9	4.5	10.5	185.07 ± 3.55	3.33 ± 0.96	0.89 ± 0.19	30%
10	3	12	183.03 ± 5.66	1.88 ± 0.57	0.92 ± 0.30	30%

Note: Doc—represents Docetaxel, and Bru—represents Brusatol.

**Table 8:** CI data of combination brusatol and docetaxel solution (50:50) in LNCaP and PC-3 cell lines.

Total Dose (nM)	CI in LNCaP Cells	CI in PC-3 Cells
5.0	0.33061	0.10535
10.0	0.59098	0.18948
20.0	0.64905	0.33766
40.0	0.76064	0.29589
80.0	0.79381	0.55168
160.0	0.95830	0.99662

## Data Availability

Data is contained within the article.

## Supplementary Materials

The following supporting information can be downloaded at: https://www.mdpi.com/article/10.3390/pharmaceutics16010114/s1, Figure S1: Values proposed for each factor to achieve particle size minimization; Figure S2: Percent viability of LNCaP and PC-3 cells treated with different concentrations of brusatol and docetaxel (50:50) in vitro at 72 h; Figure S3: Effect of brusatol and docetaxel on the growth of LNCaP and PC-3 cells; Figure S4: Combination index (CI) plot of combination brusatol and docetaxel solution at fixed ratio (50:50) of each drug for 72 h on LNCaP cells and PC-3 cells; Figure S5: Phase contrast images of LNCaP cells visualized by EVOS inverted microscope; Figure S6: Phase contrast images of PC-3 cell visualized by EVOS inverted microscope; Figure S7: Representative flow cytometry histograms of cell cycle analysis of LNCaP cells treated for 72 h; Figure S8: Representative flow cytometry histograms of cell cycle analysis of LNCaP cells treated for 120 h; Figure S9: Representative flow cytometry histograms of cell cycle analysis of PC-3 cells treated for 72 h; Figure S10: Representative flow cytometry histograms of cell cycle analysis of PC-3 cells treated for 120 h; Table S1: Cell cycle analysis data for both LNCaP and PC-3 cells following flow cytometry evaluation (*n* = 3).
