# Supplementary material for: Preparation, Optimization, and In-Vitro Evaluation of Brusatol- and Docetaxel-Loaded Nanoparticles for the Treatment of Prostate Cancer
Source: Pharmaceutics. 2024 Jan 16;16(1):114. doi: 10.3390/pharmaceutics16010114 (PMC10819281; doi:10.3390/pharmaceutics16010114)
Supplement: Supplementary file 1 [file pharmaceutics-16-00114-s001.zip › pharmaceutics-2642790-supplementary.pdf]

Supplementary Information

Preparation, Optimization, and In-Vitro Evaluation of Brusatol- and Docetaxel-Loaded Nanoparticles for the Treatment of Prostate Cancer

Tayo Alex Adekiya [1], Madison Moore [2], Michael Thomas [2], Gabriel Lake [1], Tamaro Hudson [3] and Simeon K. Adesina [1,*]

[1] Department of Pharmaceutical Sciences, College of Pharmacy, Howard University, Washington, DC 20059, USA
[2] Department of Biology, Howard University, Washington, DC 20059, USA
[3] Cancer Center, Howard University, Washington, DC 20059, USA
* Correspondence: simeon.adesina@howard.edu; Tel.: +1-202-250-5304; Fax: +1-202-806-7805

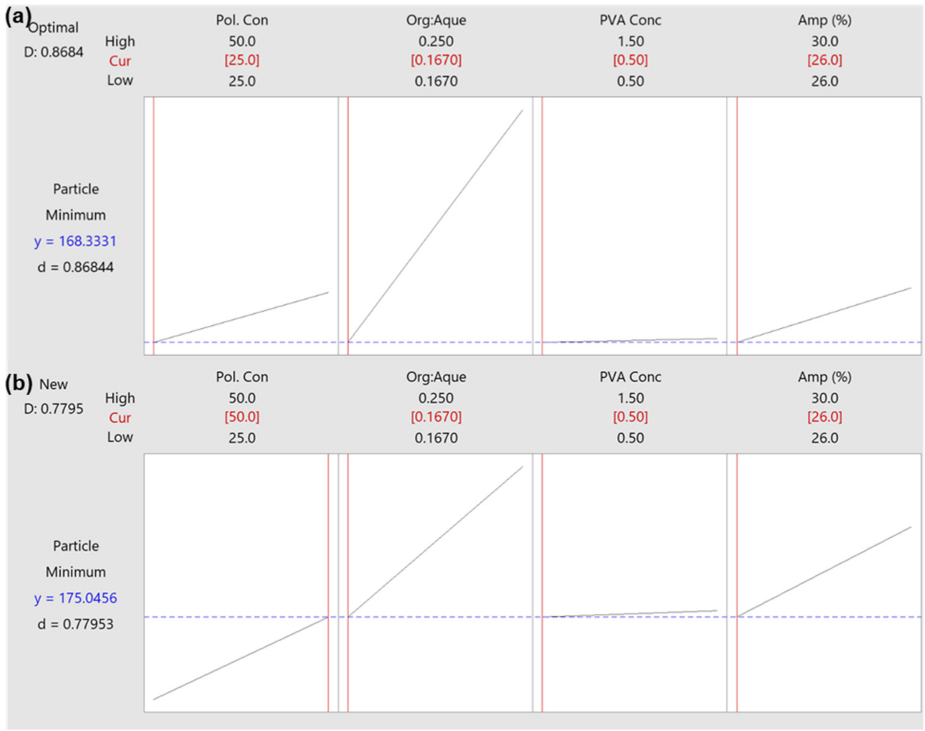

Figure S1: (a) Proposed values of each factor to achieve a particle size of 168.33 nm with composite desirability of 0.86844 (b) Proposed values of each factor to achieve a particle size of 175.05 nm with composite desirability of 0.77953.

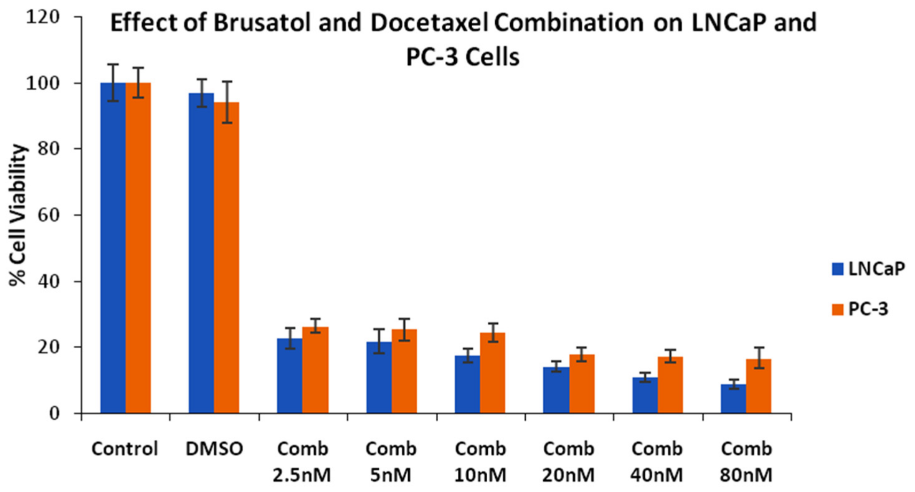

Figure S2: Percent cell viability of LNCaP and PC-3 cells treated with combination brusatol and docetaxel solution (50:50) *in vitro* at 72 hours (mean ± SD, n = 4). e.g. Comb 2.5nM represents 2.5 nM of brusatol + 2.5 nM of docetaxel. Control represents media only and DMSO represents 0.01% DMSO in media.

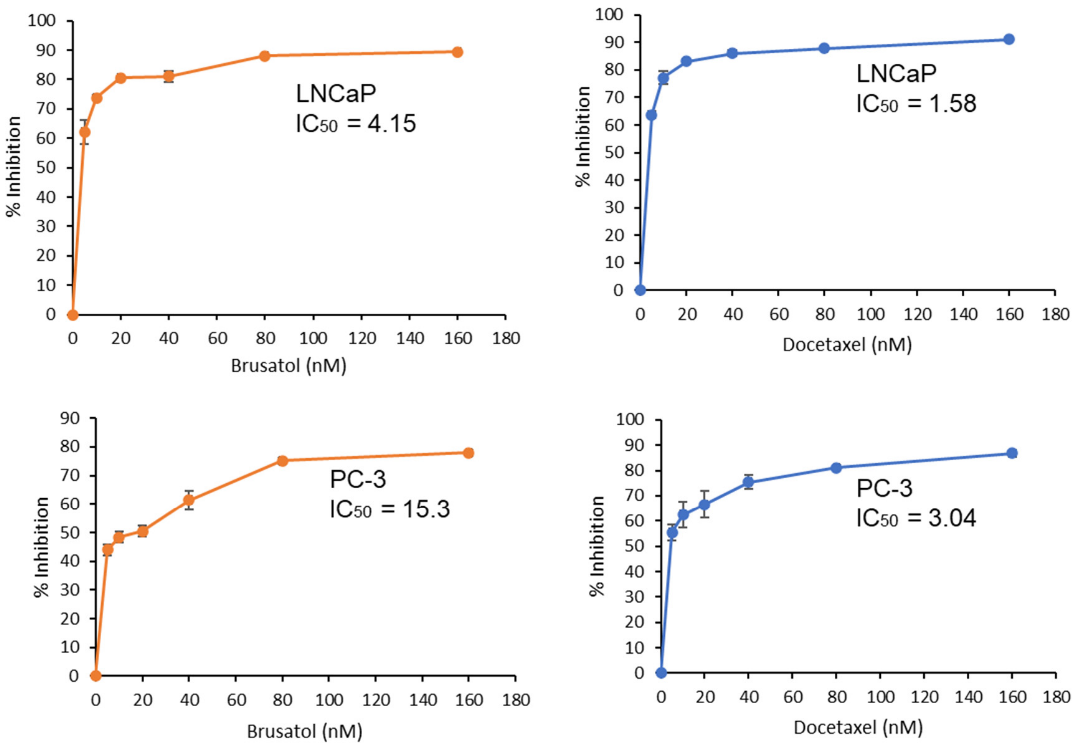

Figure S3: Effect of brusatol and docetaxel on the growth of prostate cancer cells, namely LNCaP and PC-3 cell lines. IC_{50}, half-maximal inhibitory concentration: orange color represents brusatol and blue color represent docetaxel at 72 hours.

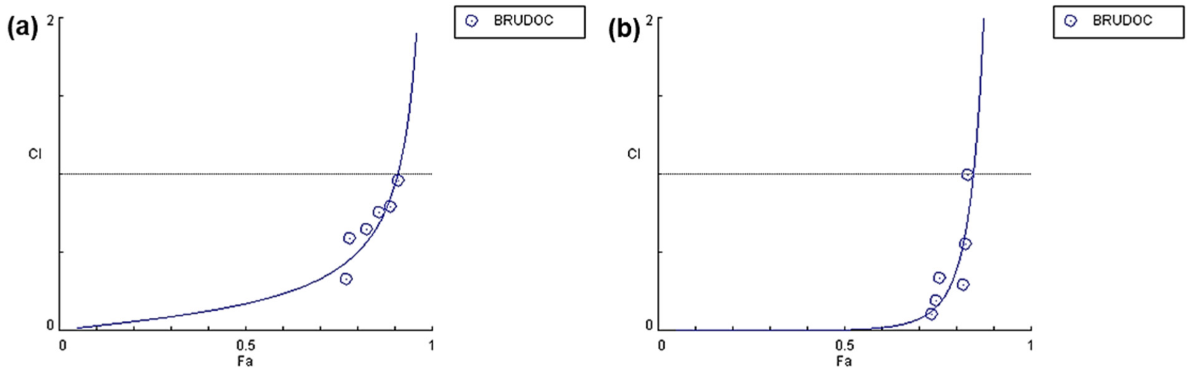

Figure S4: Combination index (CI) plot of combination brusatol and docetaxel solution at fixed ratio (50:50) of each drug for 72 hours on (a) LNCaP cells, (b) PC-3 cells. The CI was calculated by using the Chou and Talalay equation. CI values of CI<1, CI>1, and CI=1 denote synergism, antagonism, and additivity, respectively.

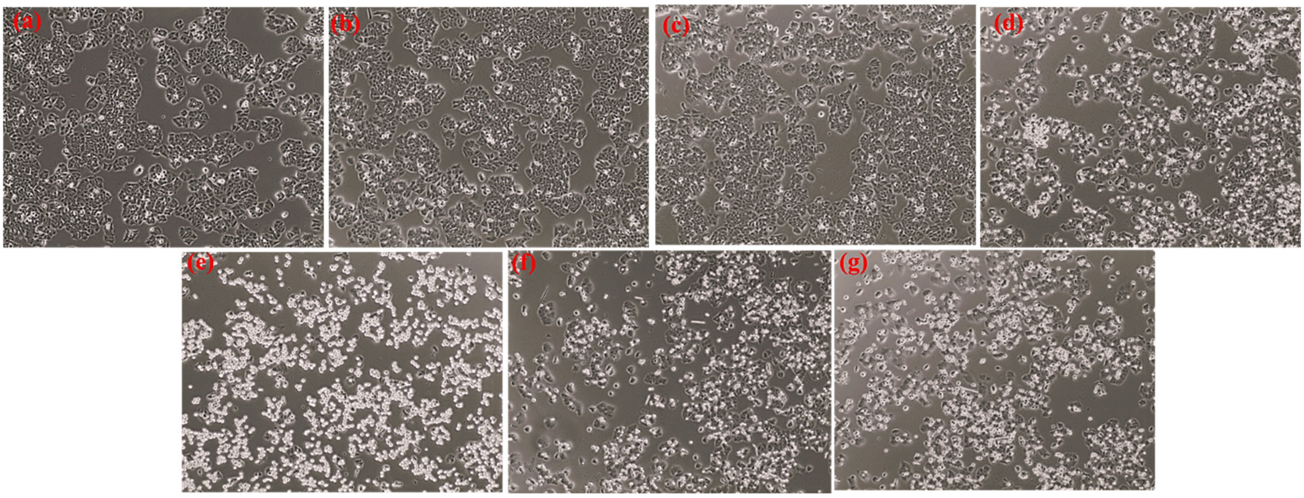

Figure S5: Phase contrast images visualized by EVOS inverted microscope (×40 magnification). showing LNCaP cells treated with(a) media only (b) DMSO solution, (c) blank-NPs-60nM, (d) brusatol-40nM, (e) docetaxel-60nM, (f) docetaxel-60nM + brusatol-40nM solution, (g) docetaxel-60nM + brusatol-40nM nanoparticles at 24h post-treatment.

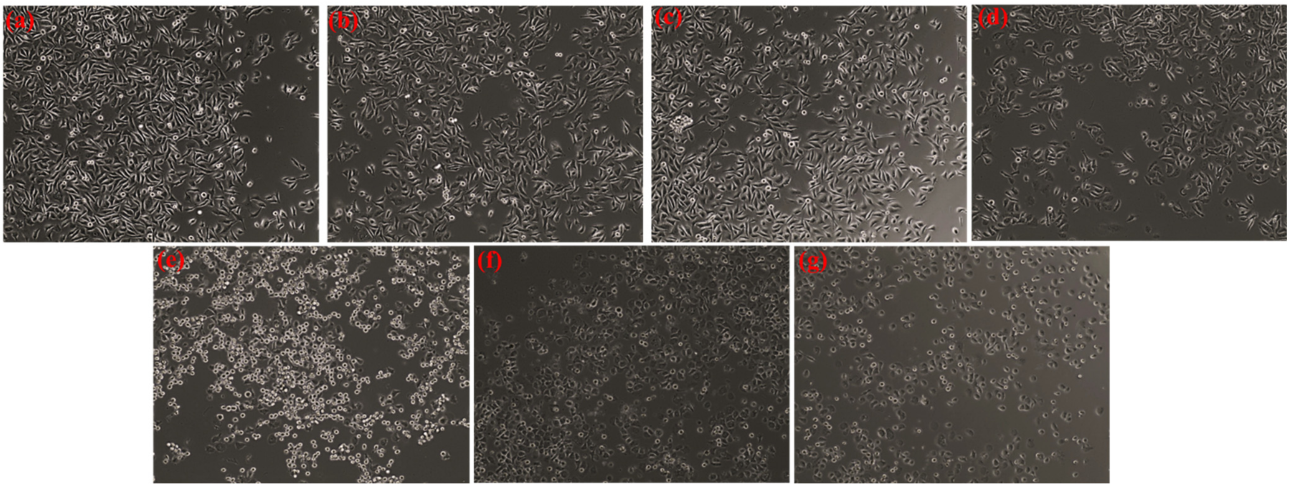

Figure S6: Phase contrast images visualized by EVOS inverted microscope (×40 magnification), showing PC-3 cells treated with (a) media only, (b) DMSO solution, (c) blank-NPs-60nM, (d) brusatol-40nM, (e) docetaxel-60nM, (f) docetaxel-60nM + brusatol-40nM solution, (g) docetaxel-60nM + brusatol-40nM nanoparticles at 24h post-treatment.

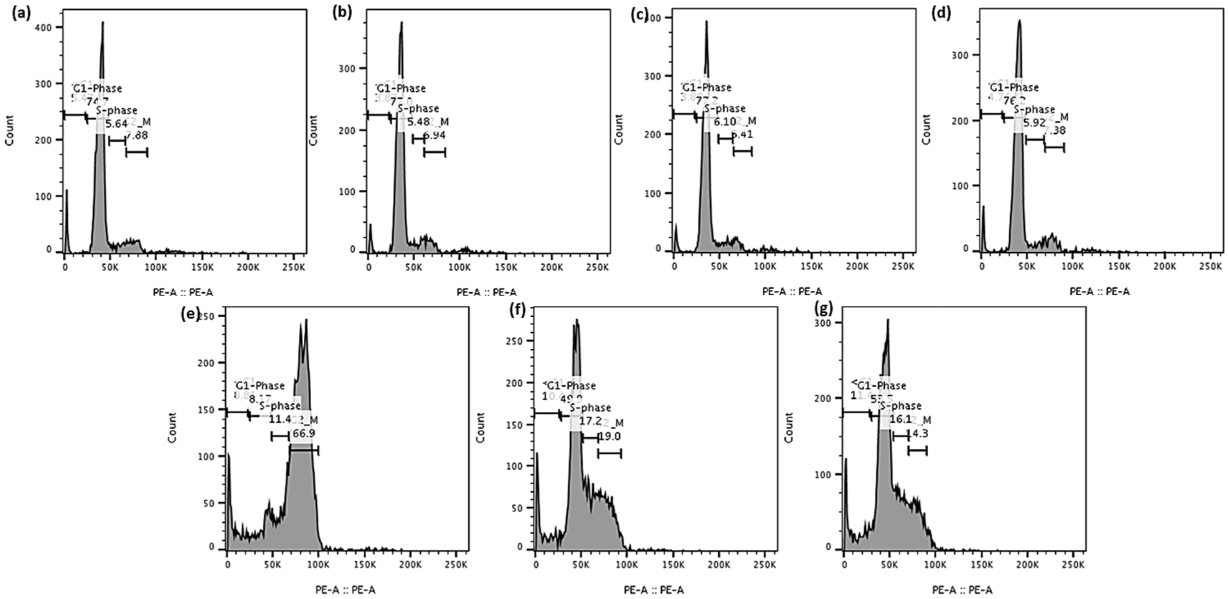

Figure S7: Representative flow cytometry histograms of cell cycle analysis of LNCaP cells treated with (a) control (b) DMSO solution, (c) blank-NPs-60nM, (d) brusatol-40nM, (e) docetaxel-60nM, (f) docetaxel-60nM + brusatol-40nM solution, (g) docetaxel-60nM + brusatol-40nM nanoparticles and incubated for 72h.

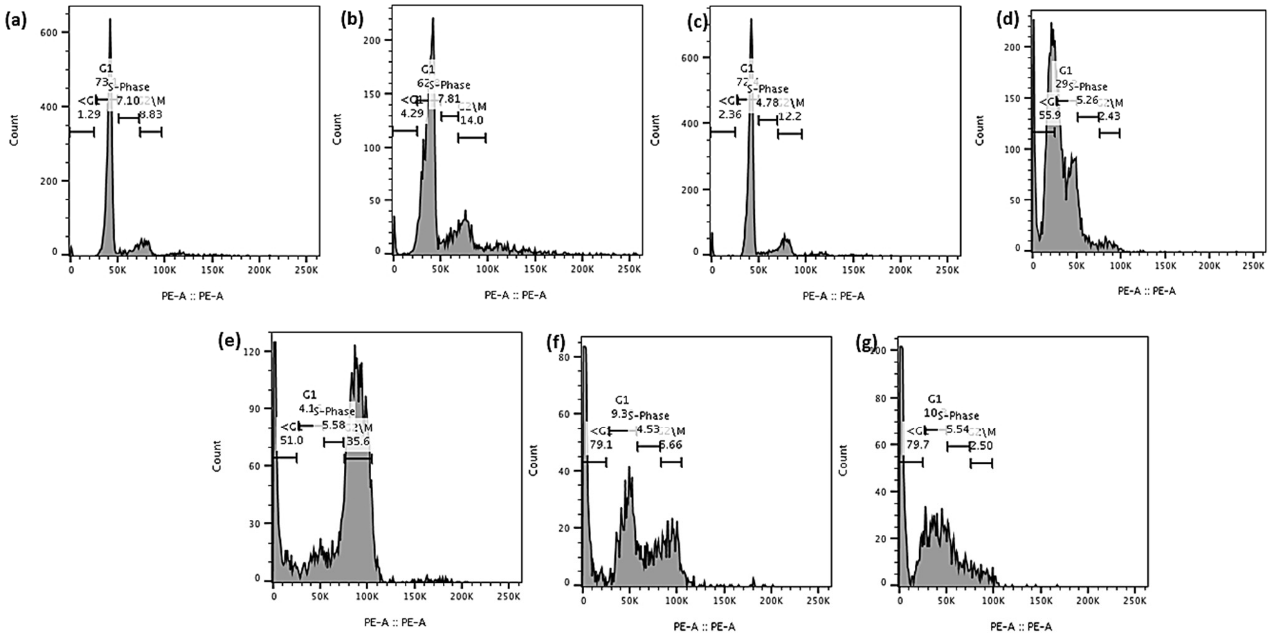

Figure S8: Representative flow cytometry histograms of cell cycle analysis of LNCaP cells treated with (a) control (b) DMSO solution, (c) blank-NPs-60nM, (d) brusatol-40nM, (e) docetaxel-60nM, (f) docetaxel-60nM + brusatol-40nM solution, (g) docetaxel-60nM + brusatol-40nM nanoparticles and incubated for 120h.

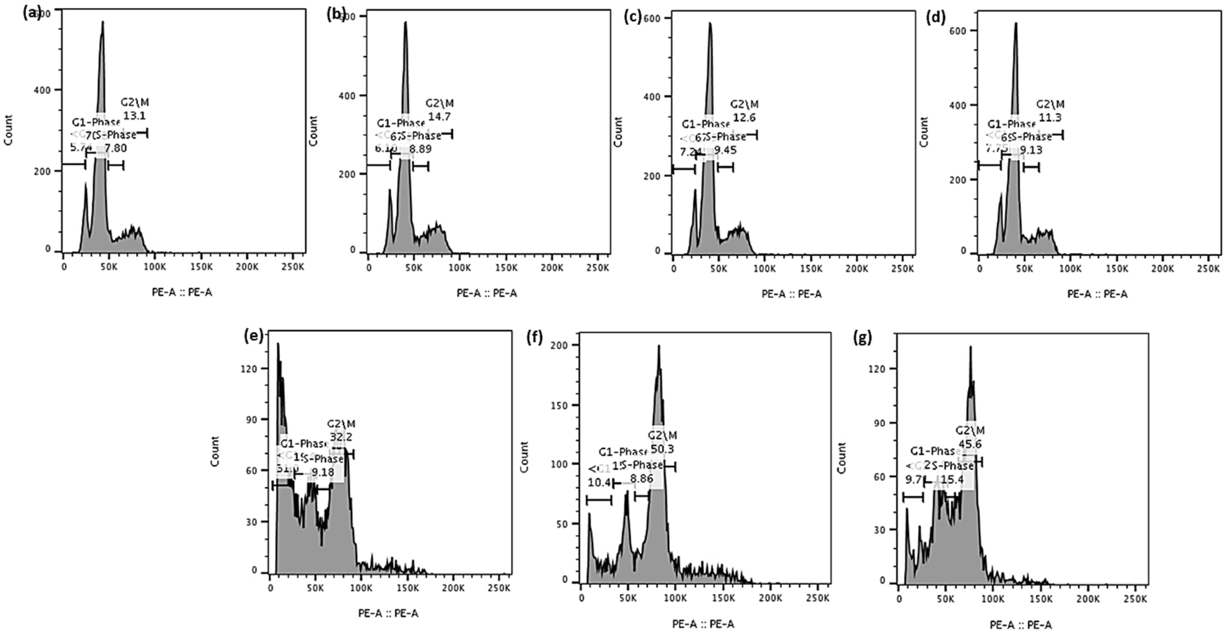

Figure S9: Representative flow cytometry histograms of cell cycle analysis of PC-3 cells treated with (a) control (b) DMSO solution, (c) blank-NPs-60nM, (d) brusatol-40nM, (e) docetaxel-60nM, (f) docetaxel-60nM + brusatol-40nM solution, (g) docetaxel-60nM + brusatol-40nM nanoparticles and incubated for 72h.

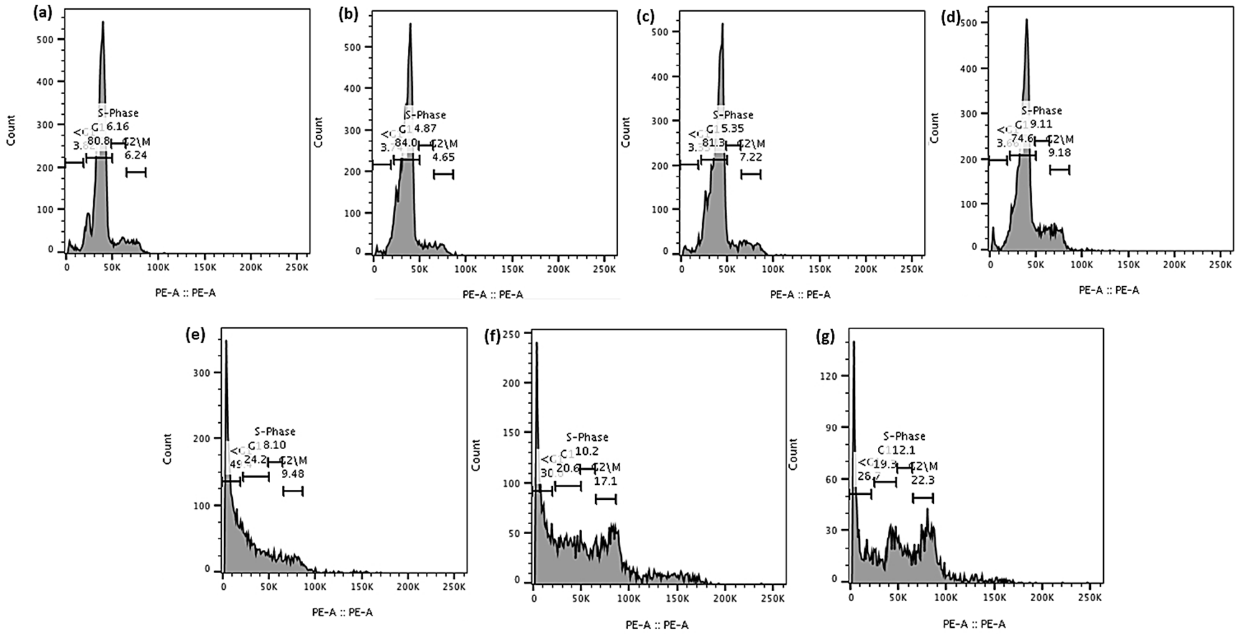

Figure S10: Representative flow cytometry histograms of cell cycle analysis of PC-3 cells treated with (a) control (b) DMSO solution, (c) blank-NPs-60nM, (d) brusatol-40nM, (e) docetaxel-60nM, (f) docetaxel-60nM + brusatol-40nM solution, (g) docetaxel-60nM + brusatol-40nM nanoparticles and incubated for 120h.

Table S1: Cell cycle analysis data for both LNCaP and PC-3 cells following flow cytometry evaluation (n = 3).

Treatment	LNCaP (%)				PC-3 (%)		
	G1	G2/M	subG1		G1	G2/M	subG1
Brusatol 40nM							
24 hour	66.6	16.6	1.02		66.1	14.6	7.94
72 hour	60.7	10.8	11.2		57	19.6	4.58
120 hour	27.6	6.04	54.2		52	18.5	11.7
Docetaxel 60nM							
24 hour	6.68	52.3	1.6		16.9	70.6	4.25
72 hour	8.17	66.9	8.81		19.8	32.2	31
120 hour	4.16	35.6	51		15.8	13.2	66.3
Brusatol 40nM + Docetaxel 60nM Solution							
24 hour	54.8	20.2	1.27		50.5	29.4	6.74
72 hour	54.1	14.2	11.7		17.8	55.6	4.84
120 hour	9.21	7.33	78.6		17.3	17.2	58.1
Brusatol 40nM+Docetaxel 60nM NP							
24 hour	56.3	18	1.75		48.3	23.7	13.5
72 hour	53.5	14.3	11.4		25.8	45.6	9.71
120 hour	10.3	2.5	79.7		10.4	23.6	56.4
